# Convergence in health care spending across counties in New York from 2007 through 2016

**DOI:** 10.1371/journal.pone.0215850

**Published:** 2019-04-24

**Authors:** Mark A. Zezza

**Affiliations:** New York State Health Foundation, New York City, New York, United States of America; Yokohama City University, JAPAN

## Abstract

**Background:**

One approach considered for reducing health care spending is to narrow the gap in spending between high- and low-spending areas. The goal would be to reduce spending in the high areas to similar levels achieved in areas that use health care more efficiently. This paper examined the degree to which high-spending areas remain high-spending and which types of service lead to convergence or divergence in spending in New York State.

**Methods:**

This analysis utilized publicly available data on county-level spending trends for the Medicare fee-for-service population from 2007 to 2016. The study applied methods previously used to evaluate changes in the regional variation of health care spending nationally to county-level data within New York.

**Results:**

The spread of health care spending converged slightly over the ten-year period analyzed. There was also evidence for regression to the mean-effects and changes in the relative rankings of spending across counties during this time. While there was strong evidence for convergence, many high-spending counties in 2007 remained high-spending in 2016. There were also differences in which services drove spending variation at the national level compared to within New York.

**Conclusions:**

These findings point to counties with consistently high spending as a potential focus for health care cost-control efforts. Moreover, efforts to reduce unwarranted variation in spending may need to be tailored to the circumstances of particular regions as there are geographic differences in which services drive spending variation. Regression to the mean effects also have important implications for the specifications of alternative provider payment models, such as accountable care organizations, which promote convergence in spending by utilizing spending targets.

## Introduction

Many studies have documented substantial variation in regional health care spending for Medicare enrollees across the United States, even after controlling for differences in local prices or the health status of populations suggesting that regional differences are largely due to differences in health care provider and patient preferences in resource utilization [1–4]. Moreover, there does not appear to be strong support for the notion that more spending leads to better quality care or better outcomes for patients; to the contrary, at least some research suggests that better outcomes are associated with areas that use health care more efficiently [5–7].

Given the high and increasing costs of health care in the United States, these findings have led policymakers to consider whether health care spending could be substantially decreased without harming population health outcomes. Research benchmarking spending across regions in the United States to more efficient areas that are associated with better health outcomes has suggested that up to 20 percent of health care delivered may be unwarranted and have questionable value to patient outcomes [5,7].

Thus, one approach for reducing health care costs could be to focus on regions that are associated with high spending. This approach would be most effective if high spending regions remain high spending over time.

Alternatively, if there is convergence in spending, whereby high-spending areas do not remain high spending, policy options targeting these areas may not be very effective.

An analysis of Medicare spending from 1992 through 2010 for hospital-referral regions (HRRs) across the country found that high-spending areas tended to remain high spending over time [8]. The analysis also looked at spending trends by type of service and found that high spending on inpatient hospital care was consistently associated with high overall spending levels. Additionally, certain services, such as home health and skilled nursing care, increasingly accounted for high overall health care spending during the time period studied. Implications from these results suggest that policymakers have an opportunity to reduce spending not only by focusing on reducing resource utilization in areas with the highest per capita spending, but also by targeting policies at specific types of services. Such services include those that are consistently associated with high spending, are becoming increasingly associated with high overall spending, and or exhibit increasing levels of variability over time.

The methods for this analysis build on statistical approaches used in prior studies for analyzing variation and convergence in health spending across regions within the entire United States, in order to take a deeper look at spending patterns within New York State. Data made publicly available by the Centers for Medicare & Medicaid Services (CMS) on county-level Medicare spending from 2007 through 2016 was used for the analysis.

Two main questions were examined: (1) was there convergence in Medicare spending across counties in New York from 2007 through 2016; and (2) which types of health care services contributed to convergence or divergence in spending? The first question can inform whether policies to achieve more efficient health spending should be targeted at specific regions in New York. The second question can inform which services should be targeted.

This study extends prior work in several ways. Almost all prior studies exploring the convergence of health care spending across geographic areas, have focused on spending across countries, with some research exploring convergence across various geographic area designations in the United States [8–10]. This study is the first that focuses on a single state. By focusing on a single state, this study has the potential to uncover local health care spending patterns that may deviate from national trends. Such findings highlight the need for policy efforts aiming to create more efficient use of health care to be aligned with the specific circumstances of different geographic areas, rather than based on national or even broad regional patterns. New York is an ideal state to examine given its relatively large size (having 62 counties) and that it has consistently ranked in the top ten relative to other states in terms of the amount of money spent on health care on a per capita basis [11]. In addition, this study used more recent data than prior work, encompassing early impacts of provisions passed along with the Patient Protection and Affordable Care Act (ACA). Certain provisions of the ACA aimed to reduce Medicare spending levels and variation, by encouraging the use of regional and national spending targets, such as through accountable care organizations (ACOs). Potential implications from the results on convergence of spending presented in this paper are also discussed in relation to some of these ACA provisions.

## Methods

### Data and sample

Medicare data for traditional fee-for-service (FFS) enrollees are available on the CMS Geographic Variation website [12]. The data are organized into annual files from 2007 through 2016 and provide information on the Medicare FFS population in each county including: the number of enrollees residing in the county; their associated Medicare FFS spending amounts by type of service; and the average risk score for enrollees in the county. The data files used for this analysis are those that encompass information for all Medicare FFS enrollees in each county, including those below age 65.

Actual spending estimates, as well as estimates standardized for differences in prices are provided on the CMS Geographic Variation files. For this analysis, the standardized estimates were used, which take into account differences in the prices paid by Medicare for services that are due to geographic variations in the cost of providing services (e.g., differences in rent and labor costs).

The data used for this analysis were also risk adjusted to take into account the differences in the average health status of Medicare FFS enrollees across counties. The risk scores reported on the Geographic Variation files are based on the CMS Hierarchical Condition Category (CMS-HCC) scores, which use diagnoses on Medicare FFS claims to measure patient co-morbidities, with higher scores going to patients with more (or more severe) comorbidities [13]. Risk adjustment for this analysis was conducted by dividing the county-level price-standardized spending estimates by the average CMS-HCC scores for the county. All spending estimates presented in the analysis are both price-adjusted and risk-adjusted.

The type of service categories used for this analysis include: inpatient hospital; skilled nursing facility (SNF); home health (HH); outpatient facility and ambulatory surgical center (ASC); evaluation and management (E&M) and procedures; imaging and tests; durable medical equipment (DME); ambulance; and Part B drugs. Part B drugs generally include those that are administered by a clinician in a physician's office or other health care setting, as opposed to drugs that are self-administered and acquired at a retail pharmacy.

On the CMS Geographic Variation files, the sum of Medicare FFS spending by type of service in a county does not necessarily add up to the total. One reason for this is that spending amounts were not reported separately for certain types of services; including care provided by community mental health centers and comprehensive outpatient rehabilitation facilities. These spending amounts were also not included in any of the reported categories of services. Additionally, some spending estimates by type of service (e.g., hospice spending) were not available for all counties due to small sample sizes and dollar amounts. The spending for these services were not analyzed separately, but were included in the total spending estimates. They were also included as “other” for the analysis decomposing the variation and convergence in total spending. Spending on “other” services was identified as the residual from the difference between total spending and the sum of spending for all specified types of services used in the analysis.

### Analytic methods

Three concepts of convergence were explored; sigma, beta and gamma convergence. Sigma convergence refers to the decreasing spread of the spending distribution across counties over time [14]. The trends of the coefficient of variation (CV) from 2007 through 2016 were examined for county-level per capita Medicare spending in order to determine if evidence for sigma convergence existed during the study period. The CV is the standard deviation of county-level spending divided by the mean of county-level spending. The following regression was used to model the relationship between the year and CV for each year (*t*):

$${CV}_{t}=\alpha +\alpha_{o}{YEAR}_{t}+\varepsilon_{t}$$

A signal for sigma convergence would be provided if *α*_*o*_< 0 [15].

Beta convergence (also known as “regression to the mean”) refers to an inverse relationship between changes in spending over time and initial spending levels [14]. The following regression specification was used to test for beta convergence:

$$\frac{1}{9}ln\left[\frac{Y_{k,2016}}{Y_{k,2007}}\right]=\alpha +\beta lnY_{k,2007}+\varepsilon_{k,2007}$$

where *Y*_*k*,2016_ and *Y*_*k*,2007_ represent per capita Medicare spending for county k during 2016 and 2007, respectively. The model regresses spending in the 2007 on the average annual growth rate in spending from 2007 through 2016. Evidence for beta convergence is provided if *β*< 0 [16,17]. Beta convergence is a necessary condition for the existence of sigma-convergence, but sigma convergence might not accompany beta convergence [14,18].

Gamma convergence refers to the stability of the relative rankings in spending across counties over time. If there is evidence of beta convergence, it may be possible that “leap-frogging” occurs; that is, initially higher spending counties are eventually overtaken in rank by initially lower spending counties. If there is a large degree of shifting in relative spending ranks of counties, it may be less effective to continuously target efforts to control spending on the initially high-spending counties. The Kendall’s index of rank concordance known as the Kendall’s W was utilized to measure gamma convergence:

$$W=\frac{12(\sum_{j=1}^{K}(R_{j}-\bar{R}{)}^{2})}{T^{2}(K^{3}-K)}$$

where *R*_*j*_ is the sum of the ranks for each county across time and $\bar{R}$ is the mean of the *R*_*j*_, *T* is the number of time periods evaluated and *K* is the number of counties [19,20]. The numerator estimates the actual variance in the ranks of the counties over time; the denominator represents the maximum possible value the variance can take, which occurs when all ranks are concordant across time. Hence, 0≤*W*≤1, with 1 representing perfect concordance in ranks over time. The closer W is to zero, the greater the extent of mobility there has been within the relative distribution of spending across counties. The W statistic was calculated using the rankings from the first two years (i.e., 2007 and 2008), then for three years and so on until for all ten years. This makes it possible to see how the concordance in rankings changes over time. The probability associated with the occurrence of the rankings being unrelated to each other was determined based on the *X*^2^ distribution (calculated as T(K-1)W) with K-1 degrees of freedom.

In order to explore which health care services contributed the most to the variation in spending across counties over time, the sigma convergence of total spending was decomposed into two factors: (1) the changing composition of total spending by type of service; and (2) the changing levels of relative variation within type of service. This approach has been used for similar analyses decomposing convergence in total health care spending at the state level [9]. The formula below was used to conduct the decomposition:

$$CV_{\mathrm{tot}}=\sum_{i=1}^{n}d_{i}c_{i}$$

where *n* is the number of types of services (10 in this case); *d*_*i*_ is the proportion of total spending due to the type of service *i*, and *c*_*i*_ is the relative concentration coefficient of the type of service *i*, which is defined as the product of the coefficient of variation for that type of service (CV_i_) and the correlation (*p*_*i*_) between the spending for the type of service and total spending. The formula was calculated for 2007 and 2016 spending to determine the degree to which each type of service contributed to the variation in total spending within a given year. The difference between the levels of relative variation in total spending by type of service between 2007 and 2016 was also analyzed to determine how much of the reduction (or increase) in the relative variation was attributable to each type of service.

## Results

### County-level per capita Medicare spending in New York from 2007 through 2016

Levels of unadjusted Medicare spending per capita, along with spending adjusted for input prices and health status, are presented for each of the 62 counties by type of service in New York for 2007 and 2016 in supplemental tables, S1A–S1J Table. Statewide total adjusted Medicare per capita spending increased from $7,416 in 2007 to $9,038 in 2016 for an average annual growth rate of 2.2 percent (S1A Table). Average annual growth rates across New York counties ranged from 0.4 percent (Cattaraugus) to 3.8 percent (Lewis) during this time. In 2007, there was about a 36 percent difference between the lowest and highest spending counties ($8,120 in Putnam compared to $5,989 in Lewis). The range between lowest and highest was slightly lower in 2016 at approximately 34 percent ($10,063 in Sullivan compared to $ 7,528 in Thompkins).

In general, the range in spending was wider when looking at specific services. In 2016, the narrowest range occurred for inpatient hospital spending (S1B Table) (about a 62 percent difference between the lowest and highest spending counties) and widest for Part B drug spending (S1J Table) (with a 463 percent difference).

### Was there convergence in health care spending across New York between 2007 and 2016?

#### Sigma convergence: The spread in spending across counties from 2007 through 2016

**Fig 1:**
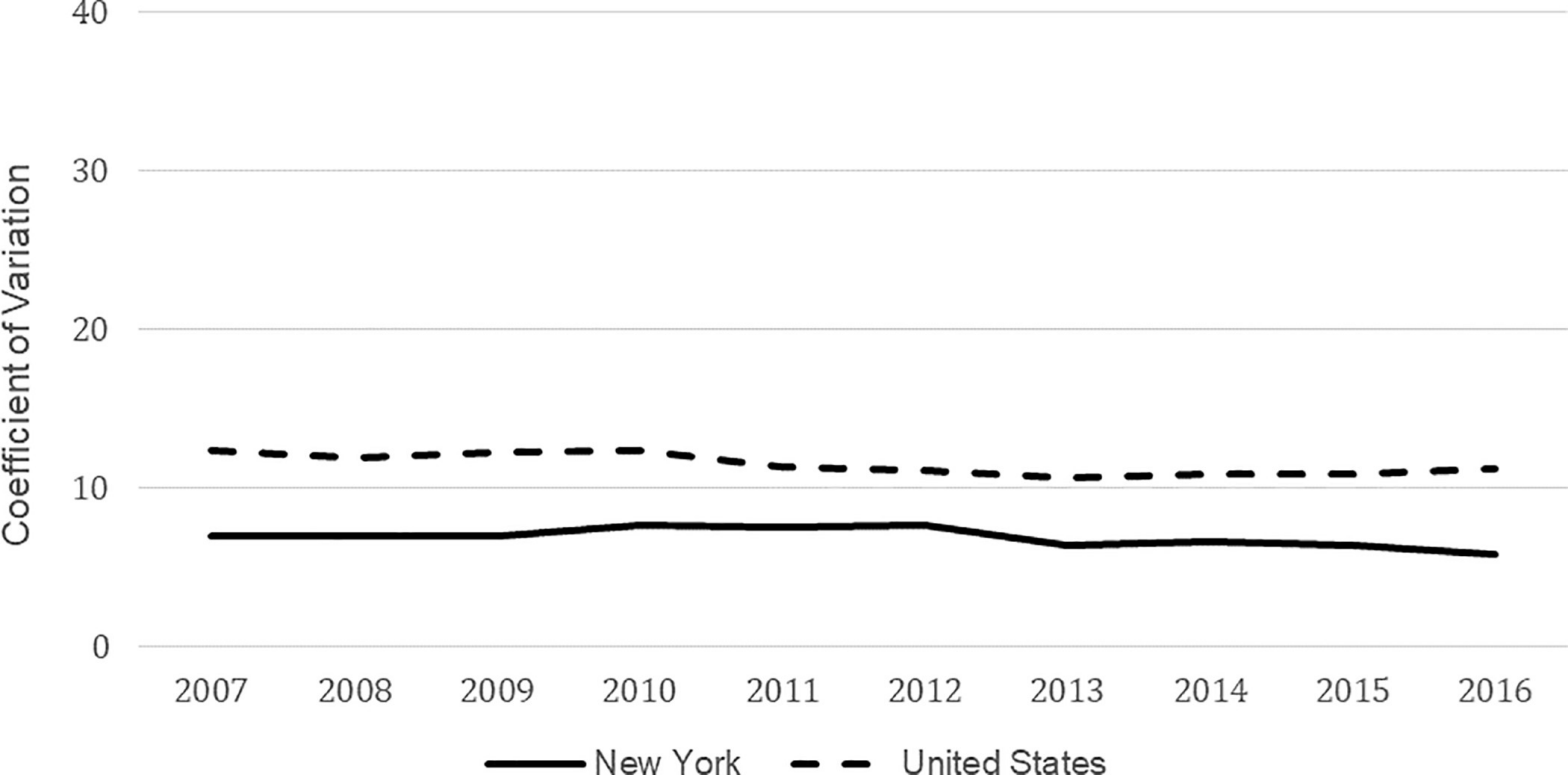
Trends in the coefficient of variation for county-level total medicare spending in New York and the United States: 2007–2016.

Fig 1 displays the trend in the coefficient of variation (CV) for total spending across counties in New York and nationally from 2007 through 2016. In both cases, evidence for sigma convergence existed as the CV trended slightly lower by the end of the time period, even though there was a period of higher CVs (i.e., evidence for divergence) from 2008 through 2012 in New York. The CV increased from 6.9 to 7.7 in 2012, before decreasing to 5.8 by 2016. The declining trend in the CV over the full time period analyzed was negative and statistically significant at the p < 0.10 level (*α*_*o*_ = -0.123, F = 4.34, p = 0.071).

The relative variation across New York was lower than across the country, which is reflective of the notion that there would be more uniformity within a state in terms of the factors that influence health care spending and utilization, such as labor costs, payment and insurance coverage policies, patient preferences, and how hospitals, physicians and other providers are organized.

#### Beta convergence: Regression to the mean in county-level spending trends

Table 1 presents the results from the regression modeling the relationship between initial spending levels in 2007 and average annual growth rates between 2007 and 2016 across counties. An inverse relationship between initial spending and growth rates was exhibited in both New York and across the United States. The results support the notion of beta convergence, reflecting regression to mean patterns in spending levels across counties. The rate of convergence during this time period was slightly higher for New York (5.4%) than the nation (3.8%).

**Table 1:** Regression results of relationship between spending levels in 2007 and average annual growth in spending between 2007 through 2016.

Statistic	New York	United States
*β* coefficient	-0.054	-0.038
Standard Error	*(0*.*010)*	*(0*.*001)*
p-value	p<0.0001	p<0.0001
Adjusted R^2^	0.34	0.24

#### Gamma convergence: The relative ranking of counties over time

Table 2 displays the results from the Kendall’s W test informing the degree of concordance in the relative rank of per capita Medicare spending across counties over time in New York and nationally. There was a consistent decline in the W statistic over time for both New York and nationally, implying an increasing amount of change in the relative ranks of county spending in years following the base year (2007). The amount of change is statistically significant at the p < 0.001 level.

**Table 2:** Kendall’s index of rank concordance for per capita medicare spending across counties in New York and nationally: 2007–2016.

Time Period	New York	United States
2007–2008	0.97	0.94
2007–2009	0.96	0.92
2007–2010	0.94	0.90
2007–2011	0.92	0.87
2007–2012	0.89	0.86
2007–2013	0.87	0.84
2007–2014	0.84	0.83
2007–2015	0.83	0.82
2007–2016	0.80	0.82

In order to help put the gamma convergence trends in perspective, an analysis of the degree to which counties remained in the same quintile of per capita Medicare spending from 2007 to 2016 was also conducted (Table 3). While about two-thirds of the counties (39 of 62) changed quintiles by 2016, there was a notable amount of persistence especially in the top and bottom quintiles. About half of the counties (7 of 13) in the highest quintile of spending in 2007 were similarly ranked in 2016. Of the 6 that migrated to a lower spending quintile, four remained in the second highest spending quintile, still in the top 40 percent of counties. There was relatively more movement from lower spending counties in 2007. Slightly less than half (6 of 13) maintained their status in the bottom quintile. Of the seven that migrated to a higher spending quintile, one county made it to the top quintile and two in the second highest.

**Table 3:** Percent of counties that were ranked in the same quintile of per capita medicare spending in both 2007 and 2016.

Quintile	Percent of Counties in Same Quintile
Lowest-20^th^ Percentile (n = 13)	46%
20^th^ - 40^th^ Percentile (n = 12)	50%
40^th^ - 60^th^ Percentile (n = 12)	25%
60^th^ - 80^th^ Percentile (n = 12)	8%
80^th^ Percentile—Highest (n = 13)	54%

### Which health care services contributed to convergence or divergence in New York between 2007 and 2016?

The coefficient of variation for per capita Medicare spending across counties in New York was about 20 percent lower in 2016 compared to 2007 (5.8 compared to 6.9). As discussed above, this finding implies sigma convergence. In order to determine how the different types of health care services contributed to the reduction in variation over time, an analysis was first conducted to examine the proportion of the variation in total spending across counties due to each type of service in 2007 and 2016. Then the changes in the composition of variation by type of service were examined from 2007 to 2016.

As shown in Table 4, most services added to the variation in total per capita Medicare spending across counties in New York in 2007 and 2016, although they generally did so to a lesser degree in 2016. E&M and procedures contributed the most amount of variation in total spending in both 2007 (42.2% = 2.92/6.92) and 2016 (35.7% = 2.29/5.80). This was largely due to E&M and procedures representing a relatively high proportion of total spending (about 18.0 percent in both years), as well as having the strongest correlation with total spending in both years (0.83 in 2007 and 0.70 in 2016). Imaging and tests explained the second largest proportion of variation in total spending in both years. Imaging and tests had the highest levels of within-service varaition during these years (CV = 36.8 in 2007 and 39.9 in 2016), as well as a relatively strong correlation with total spending.

**Table 4:** Decomposition of variation in county-level per capita total medicare FFS spending in 2007 and 2016 by type of service in New York.

	2007					2016				
Type of Service	Contribution to Variation in Total Spending *(d*_*i*_*c*_*i*_*) (% of total)*	Proportion of Total Spending *(d*_*i*_*)*	Concentration Coefficient *(c*_*i =*_ *CV*_*i*_ *p*_*i*_*)*	Coefficient of Variation *(CV*_*i*_*)*	Correlation with Total Spending *(p*_*i*_*)*	Contribution to Variation in Total Spending *(d*_*i*_*c*_*i*_*) (% of total)*	Proportion of Total Spending *(d*_*i*_*)*	Concentration Coefficient *(c*_*i =*_ *CV*_*i*_ *p*_*i*_*)*	Coefficient of Variation *(CV*_*i*_*)*	Correlation with Total Spending *(p*_*i*_*)*
Inpatient Hospital	1.61 (23.2%)	35.9%	4.48	6.5	0.69	1.45 (20.6%)	30.8%	4.71	7.8	0.60
SNF	0.92 (13.3%)	7.9%	11.75	22.5	0.52	1.12 (18.2%)	8.6%	12.98	22.9	0.57
HH	0.34 (4.8%)	3.9%	8.56	28.3	0.30	0.20 (3.0%)	3.8%	5.31	27.3	0.19
Outpatient Facility and ASC	-1.21 (-17.5%)	12.3%	-9.85	24.2	-0.41	-1.04 (-11.3%)	18.6%	-5.58	26.0	-0.21
E&M and Procedures	2.92 (42.2%)	18.0%	16.22	19.6	0.83	2.29 (35.7%)	17.5%	13.10	18.8	0.70
Imaging and Tests	2.00 (28.9%)	6.8%	29.64	36.8	0.81	1.22 (25.6%)	5.0%	24.51	39.9	0.61
DME	-0.33 (-4.7%)	3.0%	-10.75	17.0	-0.63	-0.14 (-1.6%)	2.0%	-6.94	16.8	-0.41
Ambulance	0.02 (0.3%)	1.5%	1.24	22.3	0.06	-0.04 (-0.5%)	1.5%	-2.74	17.9	-0.15
Part B Drugs	0.23 (3.4%)	3.3%	7.07	36.8	0.19	0.48 (6.8%)	4.7%	10.24	36.0	0.28
Other^1^	0.42 (6.1%)	7.4%	5.66	22.8	0.25	0.24 (3.5%)	7.5%	3.19	22.6	0.14
Total	6.92(100.0%)	100.0%				5.80 (100.0%)	100.0%			

Note

^1^ Other includes spending on inpatient rehabilitation facilities, long-term care hospitals, hospice care, community mental health centers, comprehensive outpatient rehabilitation facilities.

Inpatient hospital services comprised the largest amount of total spending 2007(35.9%) and 2016 (30.8%). However, inpatient hospital spending in New York only contributed to about a fifth of the variation in spending in both years. The disproporonatly low level of variation in total spending explained by inpatient hospital services was due to the the low variation across counties in inpatient hospital spending (6.5 in 2007 and 7.8 in 2016).

Outpatient facility and ASC actually reduced the variation in total spending across counties in both years. This was driven by a negative correlation with total spending. DME exhibited similar effects.

Table 5 presents the results of how each type of service contributed to the convergence in total spending from 2007 to 2016. These results make it clear that the sigma convergence exhibited by total spending was not consistent across all of the services. However, the decreases in variation from E&M and procedures, imaging and tests, inpatient hospital, home health, and ambulance services more than offset the increases in variation from SNF, outpatient facility and ASC, DME and Part B drugs.

**Table 5:** Decomposition of convergence in county-level per capita total medicare FFS spending from 2007 to 2016 by type of service in New York.

Type of Service	Contribution to Change in Variation in Total Spending D*(d*_*i*_*c*_*i*_*) (% of total)*	Contribution due to Change in Concentration *(d*_*i*,*2007*_)*D*(c*_*i*_*) (% of service)*	Contribution due to Change in Proportion *(c*_*i*,*2007*_)*D*(d*_*i*_*) (% of service)*	Contribution due to Interaction D*(d*_*i*_)*D*(c*_*i*_*) (% of service)*
Inpatient Hospital	-0.15 (13.8%)	0.084 (-54.5%)	-0.227 (146.8%)	-0.012 (7.7%)
SNF	0.20 (-17.4%)	0.097 (49.5%)	0.089 (45.7%)	0.009 (4.8%)
HH	-0.13 (11.6%)	-0.127 (97.2%)	-0.006 (4.6%)	0.002 (-1.7%)
Outpatient Facility and ASC	0.17 (-15.3%)	0.524 (305.5%)	-0.623 (-362.9%)	0.270 (157.4%)
E&M and Procedures	-0.62 (55.5%)	-0.562 (89.9%)	-0.078 (12.5%)	0.015 (-2.4%)
Imaging and Tests	-0.78 (69.2%)	-0.346 (44.5%)	-0.522 (67.1%)	0.090 (-11.6%)
DME	0.19 (-16.9%)	0.116 (60.8%)	0.115 (60.7%)	-0.041 (-21.5%)
Ambulance	-0.06 (5.2%)	-0.060 (102.9%)	-0.001 (1.3%)	-0.002 (-4.2%)
Part B Drugs	0.25 (-22.0%)	0.105 (42.5%)	0.098 (39.7%)	0.044 (17.8%)
Other^1^	-0.18 (16.2%)	-0.184 (100.9%)	0.003 (-1.6%)	-0.001 (0.7%)
Total	-1.13 (100.0%)			

Notes: “D()” represents the change from 2007 to 2016.

^1^ “Other” includes spending on inpatient rehabilitation facilities, long-term care hospitals, hospice care, community mental health centers, comprehensive outpatient rehabilitation facilities.

Consistent with the results explaining variation in total per capita county-level spending in 2007 and 2016, E&M and procedures and imaging and tests also explained the largest portion of the convergence in variation from 2007 to 2016 (55.5% and 69.2%, respectively). The effect of E&M and procedures was primarily driven by a concentration effect due to a decrease in the within-service variation and weakening of the correlation with total spending. The effect from imaging and tests was primarily driven by a decrease in its proportion of total spending.

While Part B drugs comprised a relatively small proportion of total spending in each year, it also exhibited the largest divergence effect. There was about an equal proportion and concentration effect that contributed to the divergence. The Part B drugs proprotion of total spending increased from 3.3% in 2007 to 4.7% in 2016. The concentration effect was largely due to an increased correlation with total spending in 2016 relative to 2007. Part B drugs also had the second most variance in county-level spending in both years.

Table 6 and Table 7 display a comparable decomposition of convergence for counties across the country. Similar to New York, inpatient hospital spending accounted for the greatest share of total spending in 2007 (33.5%) and 2016 (28.1%), but a disproportionately lower percentage of the variation in spending in each year (25.3% in 2007 and 16.1% in 2016). In contrast to New York, E&M and procedures contributed a relatively small percent of the variation in total spending (about 3.0 percent in each year). Moreover, post-acute care spending accounted for about twice as much of variation in total spending across the country, relative to NY. In 2007, SNF and HH spending accounted for 34.9% (compared to 18.1% in New York) and 44.2% in 2016 (compared to 21.2%). Also, in contrast to New York, all services added to the variation in total spending in 2007 and 2016, as each had a positive correlation with total spending in both years.

**Table 6:** Decomposition of variation in county-level per capita total medicare FFS spending in 2007 and 2016 by type of service in the United States.

	2007					2016				
Type of Service	Contribution to Variation in Total Spending *(d*_*i*_*c*_*i*_*) (% of total)*	Proportion of Total Spending *(d*_*i*_*)*	Concentration Coefficient *(c*_*i =*_ *CV*_*i*_ *p*_*i*_*)*	Coefficient of Variation *(CV*_*i*_*)*	Correlation with Total Spending *(p*_*i*_*)*	Contribution to Variation in Total Spending *(d*_*i*_*c*_*i*_*) (% of total)*	Proportion of Total Spending *(d*_*i*_*)*	Concentration Coefficient *(c*_*i =*_ *CV*_*i*_ *p*_*i*_*)*	Coefficient of Variation *(CV*_*i*_*)*	Correlation with Total Spending *(p*_*i*_*)*
Inpatient Hospital	3.12 (25.3%)	33.5%	9.32	13.7	0.68	1.81 (16.1%)	28.1%	6.44	11.3	0.57
SNF	1.52 (12.3%)	8.8%	17.29	39.2	0.44	3.40 (30.2%)	9.5%	35.67	50.8	0.70
HH	2.79 (22.6%)	5.4%	52.01	84.1	0.62	1.57 (14.0%)	5.0%	31.36	63.3	0.50
Outpatient Facility and ASC	0.89 (7.2%)	13.2%	6.74	32.8	0.21	1.31 (11.6%)	19.1%	6.85	28.3	0.24
E&M and Procedures	0.37 (3.0%)	14.7%	2.51	15.7	0.16	0.29 (2.6%)	14.5%	2.03	18.6	0.11
Imaging and Tests	0.37 (3.0%)	5.5%	6.78	28.0	0.24	0.15 (1.4%)	4.1%	3.75	30.4	0.12
DME	0.29 (2.4%)	3.5%	8.31	26.0	0.32	0.11 (1.0%)	2.2%	5.24	23.7	0.22
Ambulance	0.16 (1.3%)	1.5%	10.45	61.5	0.17	0.09 (0.8%)	1.5%	5.84	47.3	0.12
Part B Drugs	0.26 (2.1%)	3.2%	8.04	39.9	0.20	0.33 (2.9%)	4.0%	8.26	47.3	0.17
Other^1^	2.58 (20.9%)	10.7%	24.07	40.4	0.60	2.18 (19.4%)	11.9%	18.34	34.1	0.54
Total	12.35 (100.0%)	100.0%				11.26 (100.0%)	100.0%			

Note

^1^ “Other” includes spending on inpatient rehabilitation facilities, long-term care hospitals, hospice care, community mental health centers, comprehensive outpatient rehabilitation facilities.

**Table 7:** Decomposition of convergence in county-level per capita total medicare FFS spending from 2007 to 2016 by type of service in the United States.

Type of Service	Contribution to Change in Variation in Total Spending D*(d*_*i*_*c*_*i*_*) (% of total)*	Contribution due to Change in Concentration *(d*_*i*,*2007*_)*D*(c*_*i*_*) (% of service)*	Contribution due to Change in Proportion *(c*_*i*,*2007*_)*D*(d*_*i*_*) (% of service)*	Contribution due to Interaction D*(d*_*i*_)*D*(c*_*i*_*) (% of service)*
Inpatient Hospital	-1.31 (119.7%)	-0.967 (73.7%)	-0.500 (38.1%)	0.155 (-11.8%)
SNF	1.88 (-171.6%)	1.618 (86.0%)	0.128 (6.8%)	0.136 (7.2%)
HH	-1.21 (110.7%)	-1.107 (91.2%)	-0.176 (14.5%)	0.070 (-5.8%)
Outpatient Facility and ASC	0.42 (-38.1%)	0.013 (3.2%)	0.398 (95.3%)	0.006 (1.4%)
E&M and Procedures	-0.07 (6.8%)	-0.071 (95.5%)	-0.004 (5.6%)	0.001 (-1.1%)
Imaging and Tests	-0.22 (19.8%)	-0.165 (76.3%)	-0.093 (42.8%)	0.041 (-19.1%)
DME	-0.18 (16.5%)	-0.108 (60.1%)	-0.114 (63.3%)	0.042 (-23.4%)
Ambulance	-0.07 (6.4%)	-0.070 (100.0%)	0.000 (0.0%)	0.000 (0.0%
Part B Drugs	0.07 (-6.7%)	0.007 (9.2%)	0.065 (88.5%)	0.002 (2.3%
Other^1^	-0.40 (36.6%)	-0.615 (153.1%)	0.280 (-69.7%)	-0.067 (16.6%)
Total	-1.10 (100.0%)			

Notes: “D()” represents the change from 2007 to 2016.

^1^ “Other” includes spending on inpatient rehabilitation facilities, long-term care hospitals, hospice care, community mental health centers, comprehensive outpatient rehabilitation facilities.

Whereas procedures, imaging and testing services were the primary drivers of spending variation in New York during the time period analyzed, post-acute care and inpatient hospital spending were the primary drivers nationally. SNF spending actually had a divergence effect in total spending for both counties in New York and across the country. Part B drugs and outpatient facility and ASC exhibited similar effects, although on a lesser scale. These divergence effects in county-level spending across the United States were more than offset by the convergence effects in other services, primarily inpatient hospital and home health spending.

## Discussion

### Health care spending converged across counties, but there were also persistently high-spending counties

Evidence was found for convergence in per capita Medicare spending across counties in New York over the ten-year period, 2007 through 2016. This is consistent with prior findings that analyzed health care spending across the entire United States using data from the 1990s up until the passage of the ACA in 2010 [8,9]. The evidence for convergence included a slight decline in the dispersion of spending (sigma convergence), regression to the mean effects (beta convergence), and changes in the relative rankings in spending across counties (gamma convergence). While there were similar trends nationally, the evidence for convergence was stronger in New York. This is not surprising as more homogeneity in policies governing the organization and use of health care delivery systems, as well as various social and economic factors that influence the health and health care of residents, would be expected within a single state as opposed to across states.

Even though there was evidence for convergence, a substantial number of counties in New York were found to be persistently high spending over time. This finding is also consistent with prior analyses of variation across the country [8]. It also helps to validate the health care cost-control strategy of targeting high-spending areas, as long as the focus is on the persistently high-spending areas.

While there was more than a 30 percent gap in spending between the highest and lowest spending counties in New York, it is unlikely that that the gap could be completely eliminated. Research based on national spending trends suggests that up to one-fifth of health care may be wasteful [5,7]. If per capita Medicare spending could be reduced by a quarter of that (i.e., 5 percent) in just the seven counties (Nassau, New York, Orange, Putnam, Rockland, Suffolk, and Westchester) that were found to be persistently high spending from 2007 through 2016, total Medicare spending in New York could be reduced by more than two percent annually, which would have amounted to nearly $350 million in 2016. It turns out that the seven persistently high-spending counties also accounted for a disproportionally large share (about 40 percent) of Medicare FFS enrollment in New York; increasing the potential impact of focusing on these counties.

There were also counties in the lowest quintile of spending in 2007, which dramatically increased their relative ranking by 2016. These areas of high average annual growth may warrant monitoring to determine the value of the higher spending and the potential need for cost-control efforts.

### Physician services were consistent primary drivers of spending trends in New York; SNF and Part B Drugs played increasing role

A decomposition of the convergence in spending over time highlighted E&M and procedures, as well as imaging and tests as the primary drivers of spending variation trends in New York. These services were also the most highly correlated with total spending. New York has high levels of physician supply [21]. Thus it is not surprising that services prescribed and largely conducted by physicians, such as procedures, tests, and imaging, as well as E&M office visits have a key role in New York health care spending. While these services have helped narrow the variation in Medicare spending from 2007 through 2016, consideration should be given for how to further leverage their role in efforts to encourage more efficient health care spending.

Imaging and tests also exhibited the highest amount of variance across New York counties, suggesting potential benefits from increased adoption of relevant best practice guidelines. New York actually has one of the highest percentages of imaging use relative to other states; about 73 percent of Medicare beneficiaries used imaging in 2016 (rates ranged from about 61 percent to 75 percent in other states). Moreover, New York was one of only ten states to have increasing use of imaging events per 1,000 Medicare FFS enrollees from 2007 through 2016 [12]. After years of high growth in imaging use through the early 2000s, rates have declined steadily across most of the country in the last decade due to increased implementation of best practices [22,23].

It was also shown that SNF and Part B drugs had the largest divergence effects on the variance in total health spending. This was primarily due to their growing resource use, as they comprised an increased proportion of Medicare spending and an increased correlation with total spending during the study time frame. These findings suggest further exploring the value of the increased use of these services and the potential for their more efficient utilization. These findings also highlight that it is not always the service associated with the most volume or dollar amount that represent the lowest hanging fruit for health reform efforts. Part B drugs accounted for less than five percent of Medicare spending, but added the most variation to county-level per capita Medicare spending over the ten-year period analyzed.

### Spending trends in New York differ from the rest of the country

In contrast to prior studies on convergence of health care spending, this analysis focuses on variation within a single state as opposed to the entire country. A key finding is that there are differences in how the types of services factored into the changes in health care spending variation in New York, relative to all counties across the United States. For example, whereas E&M visits, procedures, imaging and tests were primary drivers of spending in New York, facility-based care such as inpatient hospital and SNF services played a substantially larger role in the rest of the country. This supports the notion that health care reforms need to be tailored to local market conditions, which raises challenges for federally run programs such as Medicare to be localized in their policies.

It should also be noted that there were weakening associations between the level of total spending and the levels of spending for most types of service from 2007 through 2016 (see Table 4 for correlations). A similar pattern was found for the United States (Table 6). This result differs from prior research, which found increasing correlations between total spending and spending by type of service from 1992 to 2010 [8]. One potential inference of this is that high health spending is becoming less associated with systemically higher resource use across the full spectrum of services offered to patients. Thus, the reason for high-spending in one geographic area, may be different than the reasons for high-spending in another area. In New York, this means that while E&M visits, procedures, imaging and tests generally play a large role in total health spending trends in New York, different services (or combinations of services) may be the primary drivers of high spending in specific areas within the state. Hence, cost-control policies likely need to be tailored to sub-state regions.

### Implications for health care provider payment reform

The evidence for convergence has implications for the development of alternative payment models (APMs) for health care providers; specifically APMs that incorporate spending targets. Such APMs include those typically associated with ACOs. ACO payment models often revolve around a prospectively set spending target based on historical spending levels of participating health care providers that are projected forward using regional (e.g., groups of counties), or even national, spending trends. Participating providers are incentivized to keep actual spending below the targets by sharing in “savings” if actual spending ends up below the targets. They may also have to share in “excess costs” should actual spending exceed targets. Hence, there are incentives for providers to deliver more efficient care. Such payment models can speed up the rate of convergence in spending by capping spending in areas with high levels to below spending targets.

Given “regression to the mean” effects found in this study, the use of participant-specific historical spending estimates along with national or regional growth rates in spending trends when developing spending targets may favor participation in areas with relatively higher historical spending as they may also be more likely to experience future spending growth lower than national or regional average levels without any intentional practice changes to lower spending. Conversely, providers may be less likely to participate in historically low spending areas expected to have higher than average future growth rates.

Studies on the early formation and performance of providers participating in the Medicare ACO program suggest that the potentially advantageous benefits of being in a high-cost area with benchmarks based on national or broad regional spending trends has not gone unnoticed. Areas having higher per capita Medicare costs were significantly more likely to have an ACO present [24]. Moreover, ACOs located in the highest spending HRR quintile were almost seven times more likely to achieve shared savings than ACOs located in an HRR in the lowest spending quintile [25].

One potential approach to help encourage participation in areas expected to have low spending and high growth rates is to adjust the spending targets in those areas to make them more obtainable. The adjustment would ideally be big enough to entice participation, but small enough to ensure Medicare spending (including shared savings payouts) would not increase relative to the status quo. This could be a short-term solution to help bolster participation. Assuming Medicare spending continues to converge, targets for providers in all areas could eventually be based upon longer-term steady-states of spending growth or even preferred growth rates in spending that are not linked to regional or national patterns (e.g., growth rates of the general economy or consumer price index).

### Limitations and future research

This analysis was limited to data from 2007 through 2016. Additional research using a longer time frame can help clarify and confirm patterns identified in this paper. For example, the decreasing trend in the CV for total spending was not consistent for each year from 2007 through 2016, raising concerns that some of the findings may be an artifact of the start and end years used for the analysis. However, prior research using data from the 1990s through 2010 also found evidence of convergence across the United States; thus, the results of this analysis might be part of a longer-term trend. As more data comes in following the implementation of the ACA, more insights on whether ACA reforms have helped speed up the rate of convergence can be gained.

This study also focuses on Medicare FFS spending. The patterns of convergence and variation by type of service may be different for non-Medicare populations such as those with employer-sponsored insurance who are generally younger, less likely to be chronically ill, and less likely to be admitted to a hospital or need post-acute care services. State and local policy-makers also have more ability to influence how health care is paid in non-Medicare markets, particularly the Medicaid and fully-insured group and non-group commercial insurance markets. However, states can also apply for Medicare waivers and innovation grants that can help them align health reform efforts across multiple payers [26]. As Medicare accounts for about a fifth of all health care spending in New York and across the country, it often acts as a market leader and can facilitate the development of system-wide health reform activities [11]. Hence, at a minimum it will be critical to understand how Medicare spending and resource patterns within New York interact with those from other payers. Research that looked at spending trends across the country has found a negative correlation between Medicare and commercial spending [4]. However, that is not the case for all states [27].

While this analysis identifies potential opportunities to create a more efficient health care system in New York, future work should investigate the explicit mechanisms through which unwarranted variation in spending are introduced, as well as specific policy solutions to reduce such variation. Such analyses would likely require more granular categorizations of types of services, as well as additional data on patient demand for health care and the supply of providers for different health care service areas.

## Conclusion

Evidence for convergence in health care spending was found. The results suggest certain combinations of regions and types of services to focus on in order to better understand and manage the variation in health care spending. The patterns of variation over time also suggest important considerations for implementing provider payment reforms with a spending target component.

## Supporting information

S1 Table Table A. Per Capita Total Medicare Spending by County in New York, Unadjusted and Adjusted Spending levels in 2007 and 2016. Table B. Per Capita Inpatient Hospital Medicare Spending by County in New York, Unadjusted and Adjusted Spending levels in 2007 and 2016. Table C. Per Capita SNF Medicare Spending by County in New York, Unadjusted and Adjusted Spending levels in 2007 and 2016. Table D. Per Capita HH Medicare Spending by County in New York, Unadjusted and Adjusted Spending levels in 2007 and 2016. Table E. Per Capita Outpatient facility & ASC Medicare Spending by County in New York, Unadjusted and Adjusted Spending levels in 2007 and 2016. Table F. Per Capita E&M and Procedures Medicare Spending by County in New York, Unadjusted and Adjusted Spending levels in 2007 and 2016. Table G. Per Capita Imaging and Tests Medicare Spending by County in New York, Unadjusted and Adjusted Spending levels in 2007 and 2016. Table H. Per Capita DME Medicare Spending by County in New York, Unadjusted and Adjusted Spending levels in 2007 and 2016. Table I. Per Capita Ambulance Medicare Spending by County in New York, Unadjusted and Adjusted Spending levels in 2007 and 2016. Table J. Per Capita Part B Drugs Medicare Spending by County in New York, Unadjusted and Adjusted Spending levels in 2007 and 2016
(DOCX)

## References

[1] Gottlieb DJ, Zhou W, Song Y, Andrews KG, Skinner JS, Sutherland JM. Prices Don’t Drive Regional Medicare Spending Variations. Health Aff (Millwood). 2010 1 28;29(3):537–43. 20110290 10.1377/hlthaff.2009.0609 PMC2919810

[2] Song Y, Skinner J, Bynum J, Sutherland J, Wennberg JE, Fisher ES. Regional Variations in Diagnostic Practices. N Engl J Med. 2010 7 1;363(1):45–53. 10.1056/NEJMsa0910881 20463332 PMC2924574

[3] Sutherland JM, Fisher ES, Skinner JS. Getting past denial—the high cost of health care in the United States. N Engl J Med. 2009 9 24;361(13):1227–30. 10.1056/NEJMp0907172 19741220

[4] Committee on Geographic Variation in Health Care Spending and Promotion of High-Value Care, Board on Health Care Services, Institute of Medicine. Variation in Health Care Spending: Target Decision Making, Not Geography [Internet]. Newhouse JP, Garber AM, Graham RP, McCoy MA, Mancher M, Kibria A, editors. Washington (DC): National Academies Press (US); 2013 [cited 2018 Nov 25]. Available from: http://www.ncbi.nlm.nih.gov/books/NBK201647/24851301

[5] Skinner J. Chapter Two—Causes and Consequences of Regional Variations in Health Care In: Pauly MV, Mcguire TG, Barros PP, editors. Handbook of Health Economics [Internet]. Elsevier; 2011 [cited 2018 Nov 18]. p. 45–93. (Handbook of Health Economics; vol. 2). Available from: http://www.sciencedirect.com/science/article/pii/B9780444535924000025

[6] Burke LA, Ryan AM. The Complex Relationship between Cost and Quality in US Health Care. AMA J Ethics. 2014 2 1;16(2):124–30. 10.1001/virtualmentor.2014.16.02.pfor1-1402 24553333

[7] Skinner JS, Fisher ES, Wennberg J. The Efficiency of Medicare. Anal Econ Aging. 2005 8 8;129–60.

[8] Chicklis C, MaCurdy T, Bhattacharya J, Shafrin J, Zaidi S, Rogers D. Regional Growth in Medicare Spending, 1992–2010. Health Serv Res. 2015 10;50(5):1574–88. 10.1111/1475-6773.12287 25676603 PMC4600362

[9] Wang Z. The convergence of health care expenditure in the US states. Health Econ. 2009 1;18(1):55–70. 10.1002/hec.1343 18273915

[10] Apergis N, Christou C, Hassapis C. Convergence in public expenditures across EU countries: evidence from club convergence. Econ Finance Res. 2013 1 1;1(1):45–59.

[11] Centers for Medicare & Medicaid Services. National Health Expenditures by State of Residence, 1991–2014 [Internet]. 2017 [cited 2018 Nov 18]. Available from: https://www.cms.gov/Research-Statistics-Data-and-Systems/Statistics-Trends-and-Reports/NationalHealthExpendData/NationalHealthAccountsStateHealthAccountsResidence.html

[12] Centers for Medicare & Medicaid Services. Medicare Geographic Variation: State/County Table—All Beneficiaries [Internet]. 2018 [cited 2018 Nov 18]. Available from: https://www.cms.gov/Research-Statistics-Data-and-Systems/Statistics-Trends-and-Reports/Medicare-Geographic-Variation/GV_PUF.html

[13] Pope GC, Kautter J, Ingbar MJ, Freeman S, Sekar R, Newhart C. Evaluation of the CMS-HCC Risk Adjustment Model [Internet]. 2011 3 Available from: https://www.cms.gov/Medicare/Health-Plans/MedicareAdvtgSpecRateStats/downloads/Evaluation_Risk_Adj_Model_2011.pdf

[14] Sala-i-Martin XX. Regional cohesion: Evidence and theories of regional growth and convergence. Eur Econ Rev. 1996 6 1;40:1325–52.

[15] Kułyk P, Augustowski Ł. Convergence Of Health Expenditure In Eu 12 And V4 States. Copernic J Finance Account. 2017;6(2):33–43.

[16] Pan J, Wang P, Qin X, Zhang S. Disparity and Convergence: Chinese Provincial Government Health Expenditures. PLOS ONE. 2013 8 19;8(8):e71474 10.1371/journal.pone.0071474 23977049 PMC3747206

[17] Goli S, Moradhvaj, Chakravorty S, Rammohan A. World health status 1950–2015: Converging or diverging. PLOS ONE. 2019 3 19;14(3):e0213139 10.1371/journal.pone.0213139 30889208 PMC6424455

[18] Young AT, Higgins MJ, Levy D. Sigma Convergence versus Beta Convergence: Evidence from U.S. County-Level Data. J Money Credit Bank. 2008 8;40(5):1083–93.

[19] Boyle G, McCarthy T. A simple Measure of B Convergence. Oxf Bull Econ Stat. 1997;59(2):257–64.

[20] Jha R. Growth, Inequality and Poverty in India. Econ Polictical Wkly. 2000;921–8.

[21] Association of American Medical Colleges. 2017 State Physician Workforce Data Report [Internet]. 2017 Nov. Available from: https://members.aamc.org/eweb/upload/2017%20State%20Physician%20Workforce%20Data%20Report.pdf

[22] ABIM Foundation. Choosing Wisely: Clinician Lists [Internet]. [cited 2019 Mar 31]. Available from: https://www.choosingwisely.org/clinician-lists/

[23] Medicare Payment Advisory Commission. Report to the Congress: Medicare Payment Policy [Internet]. 2017 Mar [cited 2019 Mar 31]. Available from: http://www.medpac.gov/docs/default-source/reports/mar17_medpac_ch4.pdf?sfvrsn=0

[24] Lewis VA, Colla CH, Carluzzo KL, Kler SE, Fisher ES. Accountable Care Organizations in the United States: market and demographic factors associated with formation. Health Serv Res. 2013 12;48(6 Pt 1):1840–58. 24117222 10.1111/1475-6773.12102 PMC3876396

[25] Heiser S, Colla CH, Fisher ES. Unpacking The Medicare Shared Savings Proposed Rule: Geography And Policy [Internet]. Health Affairs Blog. 2015 [cited 2018 Nov 26]. Available from: https://www.healthaffairs.org/do/10.1377/hblog20150122.044093/full/

[26] Centers for Medicare & Medicaid Services. State Innovation Models Initiative: General Information | Center for Medicare & Medicaid Innovation [Internet]. [cited 2019 Mar 31]. Available from: https://innovation.cms.gov/initiatives/state-innovations/

[27] Franzini L, Mikhail OI, Zezza M, Chan I, Shen S, Smith JD. Comparing variation in Medicare and private insurance spending in Texas. Am J Manag Care. 2011 12 1;17(12):e488–495. 22216873

