# Supplementary material for: Convergence in health care spending across counties in New York from 2007 through 2016
Source: PLoS One. 2019 Apr 24;14(4):e0215850. doi: 10.1371/journal.pone.0215850 (PMC6481841; doi:10.1371/journal.pone.0215850)
Supplement: S1 Table — Table A. Per Capita Total Medicare Spending by County in New York, Unadjusted and Adjusted Spending levels in 2007 and 2016. Table B. Per Capita Inpatient Hospital Medicare Spending by County in New York, Unadjusted and Adjusted Spending levels in 2007 and 2016. Table C. Per Capita SNF Medicare Spending by County in New York, Unadjusted and Adjusted Spending levels in 2007 and 2016. Table D. Per Capita HH Medicare Spending by County in New York, Unadjusted and Adjusted Spending levels in 2007 and 2016. Table E. Per Capita Outpatient facility & ASC Medicare Spending by County in New York, Unadjusted and Adjusted Spending levels in 2007 and 2016. Table F. Per Capita E&M and Procedures Medicare Spending by County in New York, Unadjusted and Adjusted Spending levels in 2007 and 2016. Table G. Per Capita Imaging and Tests Medicare Spending by County in New York, Unadjusted and Adjusted Spending levels in 2007 and 2016. Table H. Per Capita DME Medicare Spending by County in New York, Unadjusted and Adjusted Spending levels in 2007 and 2016. Table I. Per Capita Ambulance Medicare Spending by County in New York, Unadjusted and Adjusted Spending levels in 2007 and 2016. Table J. Per Capita Part B Drugs Medicare Spending by County in New York, Unadjusted and Adjusted Spending levels in 2007 and 2016 (DOCX) [file pone.0215850.s001.docx]

**S1A Table. Per Capita Total Medicare Spending by County in New York, Unadjusted and Adjusted Spending levels in 2007 and 2016**

|  | **Unadjusted** | |  | **Adjusted Spending*** | | **Average Annual Percent Change in Adjusted Spending: 2007-2016** |
| --- | --- | --- | --- | --- | --- | --- |
| **County** | **2007** | **2016** |  | **2007** | **2016** |  |
| STATE TOTAL | $9,577 | $11,762 |  | $7,416 | $9,038 | 2.2% |
| Albany | $6,942 | $9,066 |  | $6,927 | $8,746 | 2.6% |
| Allegany | $6,438 | $7,909 |  | $6,762 | $7,998 | 1.9% |
| Bronx | $13,273 | $15,816 |  | $7,402 | $8,592 | 1.7% |
| Broome | $6,462 | $8,399 |  | $6,938 | $8,606 | 2.4% |
| Cattaraugus | $7,170 | $7,817 |  | $7,312 | $7,628 | 0.5% |
| Cayuga | $6,482 | $9,182 |  | $6,895 | $8,963 | 3.0% |
| Chautauqua | $6,505 | $8,515 |  | $6,671 | $8,588 | 2.8% |
| Chemung | $6,484 | $9,014 |  | $6,775 | $8,622 | 2.7% |
| Chenango | $5,667 | $7,784 |  | $6,216 | $7,929 | 2.7% |
| Clinton | $6,761 | $9,626 |  | $6,938 | $8,839 | 2.7% |
| Columbia | $6,986 | $9,055 |  | $7,032 | $8,686 | 2.4% |
| Cortland | $6,663 | $8,776 |  | $6,968 | $8,163 | 1.8% |
| Delaware | $6,900 | $9,155 |  | $7,173 | $9,103 | 2.7% |
| Dutchess | $8,333 | $10,697 |  | $7,460 | $8,938 | 2.0% |
| Erie | $7,010 | $9,340 |  | $6,907 | $8,221 | 2.0% |
| Essex | $6,431 | $8,543 |  | $7,024 | $9,021 | 2.8% |
| Franklin | $6,155 | $8,028 |  | $6,316 | $7,611 | 2.1% |
| Fulton | $6,200 | $9,294 |  | $6,440 | $8,796 | 3.5% |
| Genesee | $6,879 | $8,740 |  | $6,621 | $7,855 | 1.9% |
| Greene | $6,839 | $9,376 |  | $6,849 | $8,768 | 2.8% |
| Hamilton | $6,406 | $7,128 |  | $6,857 | $8,306 | 2.2% |
| Herkimer | $7,232 | $8,873 |  | $7,378 | $8,673 | 1.8% |
| Jefferson | $6,148 | $9,194 |  | $6,250 | $8,338 | 3.3% |
| Kings | $13,923 | $15,525 |  | $7,515 | $8,845 | 1.8% |
| Lewis | $5,534 | $8,591 |  | $5,989 | $8,681 | 4.2% |
| Livingston | $6,342 | $8,067 |  | $6,186 | $7,937 | 2.8% |
| Madison | $6,360 | $8,073 |  | $6,860 | $8,419 | 2.3% |
| Monroe | $7,470 | $9,837 |  | $6,995 | $8,539 | 2.2% |
| Montgomery | $7,013 | $9,098 |  | $6,945 | $8,637 | 2.5% |
| Nassau | $10,881 | $12,769 |  | $8,008 | $9,695 | 2.1% |
| New York | $11,395 | $12,737 |  | $7,598 | $9,190 | 2.1% |
| Niagara | $7,540 | $9,368 |  | $7,175 | $8,608 | 2.0% |
| Oneida | $6,854 | $8,798 |  | $7,124 | $8,538 | 2.0% |
| Onondaga | $6,928 | $9,342 |  | $6,941 | $8,902 | 2.8% |
| Ontario | $6,226 | $8,288 |  | $6,469 | $8,681 | 3.3% |
| Orange | $9,357 | $11,352 |  | $7,561 | $9,010 | 2.0% |
| Orleans | $6,078 | $9,332 |  | $6,605 | $8,563 | 2.9% |
| Oswego | $7,004 | $9,235 |  | $7,307 | $9,102 | 2.5% |
| Otsego | $6,117 | $8,992 |  | $6,506 | $9,029 | 3.7% |
| Putnam | $9,189 | $11,128 |  | $8,120 | $9,594 | 1.9% |
| Queens | $11,760 | $13,723 |  | $7,432 | $8,793 | 1.9% |
| Rensselaer | $7,317 | $9,184 |  | $7,029 | $8,829 | 2.6% |
| Richmond | $11,432 | $12,609 |  | $7,962 | $8,944 | 1.3% |
| Rockland | $10,041 | $12,235 |  | $7,936 | $9,637 | 2.2% |
| Saratoga | $6,396 | $8,157 |  | $6,897 | $8,839 | 2.8% |
| Schenectady | $6,518 | $8,609 |  | $6,719 | $8,413 | 2.5% |
| Schoharie | $6,135 | $8,588 |  | $6,593 | $8,771 | 3.2% |
| Schuyler | $5,744 | $8,084 |  | $6,681 | $8,346 | 2.5% |
| Seneca | $6,238 | $8,621 |  | $6,775 | $9,148 | 3.4% |
| St. Lawrence | $6,473 | $8,582 |  | $6,824 | $8,415 | 2.4% |
| Steuben | $6,106 | $7,509 |  | $6,505 | $7,831 | 2.1% |
| Suffolk | $9,939 | $13,007 |  | $7,874 | $10,063 | 2.8% |
| Sullivan | $8,256 | $9,810 |  | $7,285 | $8,120 | 1.2% |
| Tioga | $6,136 | $7,585 |  | $7,158 | $8,099 | 1.4% |
| Tompkins | $5,801 | $6,815 |  | $6,502 | $7,528 | 1.6% |
| Ulster | $7,692 | $9,640 |  | $7,140 | $8,993 | 2.6% |
| Warren | $6,541 | $7,996 |  | $7,149 | $8,719 | 2.2% |
| Washington | $6,606 | $8,292 |  | $6,955 | $8,409 | 2.1% |
| Wayne | $6,353 | $9,436 |  | $6,678 | $8,724 | 3.0% |
| Westchester | $10,286 | $12,510 |  | $7,761 | $9,488 | 2.3% |
| Wyoming | $5,899 | $8,411 |  | $6,349 | $8,288 | 3.0% |
| Yates | $5,476 | $7,395 |  | $6,117 | $8,490 | 3.7% |

Notes: * Adjusted for geographic differences in prices (i.e., the cost of cost of providing services) and health status using the average CMS-HCC score for Medicare enrollees in each county.

**S1B Table. Per Capita Inpatient Hospital Medicare Spending by County in New York, Unadjusted and Adjusted Spending levels in 2007 and 2016**

|  | **Unadjusted** | |  | **Adjusted Spending*** | | **Average Annual Percent Change in Adjusted Spending: 2007-2016** |
| --- | --- | --- | --- | --- | --- | --- |
| **County** | **2007** | **2016** |  | **2007** | **2016** |  |
| STATE TOTAL | $4,084 | $4,447 |  | $2,600 | $2,715 | 0.5% |
| Albany | $2,581 | $3,303 |  | $2,360 | $2,820 | 2.0% |
| Allegany | $2,743 | $2,895 |  | $2,648 | $2,498 | -0.6% |
| Bronx | $6,945 | $7,675 |  | $2,884 | $2,928 | 0.2% |
| Broome | $2,368 | $2,915 |  | $2,331 | $2,731 | 1.8% |
| Cattaraugus | $2,949 | $2,645 |  | $2,785 | $2,282 | -2.2% |
| Cayuga | $2,645 | $3,578 |  | $2,536 | $2,964 | 1.7% |
| Chautauqua | $2,323 | $2,808 |  | $2,220 | $2,512 | 1.4% |
| Chemung | $2,628 | $3,176 |  | $2,612 | $2,749 | 0.6% |
| Chenango | $2,276 | $2,883 |  | $2,238 | $2,459 | 1.1% |
| Clinton | $2,748 | $3,919 |  | $2,594 | $2,844 | 1.0% |
| Columbia | $2,591 | $3,127 |  | $2,377 | $2,656 | 1.2% |
| Cortland | $2,692 | $3,260 |  | $2,607 | $2,685 | 0.3% |
| Delaware | $2,787 | $3,120 |  | $2,514 | $2,538 | 0.1% |
| Dutchess | $3,353 | $4,010 |  | $2,557 | $2,831 | 1.1% |
| Erie | $2,760 | $3,548 |  | $2,397 | $2,550 | 0.7% |
| Essex | $2,499 | $2,624 |  | $2,452 | $2,298 | -0.7% |
| Franklin | $2,576 | $3,106 |  | $2,381 | $2,418 | 0.2% |
| Fulton | $2,447 | $3,503 |  | $2,379 | $2,892 | 2.2% |
| Genesee | $3,010 | $3,224 |  | $2,529 | $2,405 | -0.6% |
| Greene | $2,589 | $3,288 |  | $2,361 | $2,771 | 1.8% |
| Hamilton | $2,652 | $2,346 |  | $2,271 | $2,494 | 1.0% |
| Herkimer | $2,973 | $3,058 |  | $2,740 | $2,514 | -1.0% |
| Jefferson | $2,316 | $3,608 |  | $2,145 | $2,551 | 1.9% |
| Kings | $6,728 | $6,464 |  | $2,716 | $2,672 | -0.2% |
| Lewis | $2,361 | $3,251 |  | $2,350 | $2,687 | 1.5% |
| Livingston | $2,632 | $2,917 |  | $2,238 | $2,315 | 0.4% |
| Madison | $2,496 | $2,773 |  | $2,401 | $2,504 | 0.5% |
| Monroe | $3,098 | $3,928 |  | $2,502 | $2,844 | 1.4% |
| Montgomery | $2,642 | $3,314 |  | $2,399 | $2,749 | 1.5% |
| Nassau | $4,234 | $4,368 |  | $2,653 | $2,667 | 0.1% |
| New York | $5,222 | $5,117 |  | $2,642 | $2,692 | 0.2% |
| Niagara | $3,123 | $3,558 |  | $2,612 | $2,697 | 0.4% |
| Oneida | $2,712 | $2,894 |  | $2,630 | $2,535 | -0.4% |
| Onondaga | $2,495 | $3,303 |  | $2,180 | $2,676 | 2.3% |
| Ontario | $2,515 | $3,011 |  | $2,366 | $2,853 | 2.1% |
| Orange | $3,921 | $4,168 |  | $2,697 | $2,836 | 0.6% |
| Orleans | $2,619 | $3,806 |  | $2,642 | $2,916 | 1.1% |
| Oswego | $2,775 | $3,222 |  | $2,621 | $2,743 | 0.5% |
| Otsego | $2,626 | $3,578 |  | $2,320 | $2,779 | 2.0% |
| Putnam | $3,562 | $4,104 |  | $2,733 | $3,030 | 1.2% |
| Queens | $5,380 | $5,431 |  | $2,647 | $2,561 | -0.4% |
| Rensselaer | $2,771 | $3,228 |  | $2,447 | $2,786 | 1.5% |
| Richmond | $5,037 | $4,885 |  | $2,781 | $2,644 | -0.6% |
| Rockland | $3,745 | $4,048 |  | $2,549 | $2,735 | 0.8% |
| Saratoga | $2,363 | $2,613 |  | $2,400 | $2,604 | 0.9% |
| Schenectady | $2,456 | $3,063 |  | $2,398 | $2,645 | 1.1% |
| Schoharie | $2,628 | $3,356 |  | $2,379 | $2,701 | 1.4% |
| Schuyler | $2,226 | $2,697 |  | $2,504 | $2,495 | 0.0% |
| Seneca | $2,516 | $3,128 |  | $2,492 | $2,987 | 2.0% |
| St. Lawrence | $2,720 | $3,233 |  | $2,588 | $2,633 | 0.2% |
| Steuben | $2,552 | $2,654 |  | $2,510 | $2,421 | -0.4% |
| Suffolk | $3,835 | $4,579 |  | $2,649 | $2,925 | 1.1% |
| Sullivan | $3,606 | $3,710 |  | $2,716 | $2,549 | -0.7% |
| Tioga | $2,342 | $2,708 |  | $2,483 | $2,581 | 0.4% |
| Tompkins | $2,229 | $2,187 |  | $2,322 | $2,082 | -1.2% |
| Ulster | $3,226 | $3,392 |  | $2,629 | $2,737 | 0.4% |
| Warren | $2,483 | $2,488 |  | $2,538 | $2,512 | -0.1% |
| Washington | $2,552 | $2,738 |  | $2,489 | $2,555 | 0.3% |
| Wayne | $2,747 | $4,045 |  | $2,606 | $3,375 | 2.9% |
| Westchester | $4,128 | $4,496 |  | $2,606 | $2,811 | 0.8% |
| Wyoming | $2,529 | $3,286 |  | $2,416 | $2,696 | 1.2% |
| Yates | $2,287 | $2,376 |  | $2,356 | $2,453 | 0.5% |

Notes: * Adjusted for geographic differences in prices (i.e., the cost of cost of providing services) and health status using the average CMS-HCC score for Medicare enrollees in each county.

**S1C Table. Per Capita SNF Medicare Spending by County in New York, Unadjusted and Adjusted Spending levels in 2007 and 2016**

|  | **Unadjusted** | |  | **Adjusted Spending*** | | **Average Annual Percent Change in Adjusted Spending: 2007-2016** |
| --- | --- | --- | --- | --- | --- | --- |
| **County** | **2007** | **2016** |  | **2007** | **2016** |  |
| STATE TOTAL | $698 | $1,045 |  | $581 | $868 | 4.6% |
| Albany | $385 | $525 |  | $424 | $585 | 3.7% |
| Allegany | $507 | $669 |  | $609 | $786 | 2.9% |
| Bronx | $1,066 | $1,606 |  | $684 | $1,056 | 4.9% |
| Broome | $478 | $556 |  | $562 | $656 | 1.7% |
| Cattaraugus | $697 | $554 |  | $799 | $607 | -3.0% |
| Cayuga | $423 | $671 |  | $510 | $770 | 4.7% |
| Chautauqua | $472 | $624 |  | $539 | $707 | 3.1% |
| Chemung | $374 | $727 |  | $440 | $791 | 6.7% |
| Chenango | $393 | $590 |  | $499 | $712 | 4.0% |
| Clinton | $433 | $607 |  | $512 | $713 | 3.8% |
| Columbia | $598 | $804 |  | $686 | $886 | 2.9% |
| Cortland | $490 | $519 |  | $575 | $562 | -0.3% |
| Delaware | $520 | $849 |  | $635 | $1,043 | 5.7% |
| Dutchess | $734 | $962 |  | $699 | $862 | 2.4% |
| Erie | $667 | $774 |  | $716 | $742 | 0.4% |
| Essex | $599 | $768 |  | $741 | $979 | 3.1% |
| Franklin | $286 | $309 |  | $347 | $360 | 0.4% |
| Fulton | $319 | $614 |  | $374 | $658 | 6.5% |
| Genesee | $605 | $548 |  | $676 | $576 | -1.8% |
| Greene | $440 | $848 |  | $498 | $921 | 7.1% |
| Hamilton | $241 | $391 |  | $312 | $503 | 5.4% |
| Herkimer | $753 | $929 |  | $879 | $1,045 | 1.9% |
| Jefferson | $300 | $573 |  | $350 | $634 | 6.8% |
| Kings | $786 | $1,495 |  | $473 | $956 | 8.1% |
| Lewis | $297 | $593 |  | $375 | $723 | 7.6% |
| Livingston | $404 | $451 |  | $448 | $534 | 2.0% |
| Madison | $475 | $784 |  | $554 | $899 | 5.5% |
| Monroe | $478 | $506 |  | $505 | $522 | 0.4% |
| Montgomery | $418 | $661 |  | $474 | $721 | 4.8% |
| Nassau | $835 | $1,159 |  | $622 | $920 | 4.4% |
| New York | $625 | $966 |  | $455 | $773 | 6.1% |
| Niagara | $622 | $714 |  | $653 | $709 | 0.9% |
| Oneida | $600 | $789 |  | $698 | $846 | 2.2% |
| Onondaga | $571 | $808 |  | $605 | $840 | 3.7% |
| Ontario | $516 | $577 |  | $588 | $679 | 1.6% |
| Orange | $681 | $1,118 |  | $584 | $890 | 4.8% |
| Orleans | $327 | $779 |  | $388 | $853 | 9.1% |
| Oswego | $544 | $706 |  | $596 | $758 | 2.7% |
| Otsego | $309 | $632 |  | $400 | $801 | 8.0% |
| Putnam | $874 | $1,123 |  | $740 | $988 | 3.3% |
| Queens | $965 | $1,428 |  | $647 | $1,007 | 5.0% |
| Rensselaer | $514 | $624 |  | $540 | $681 | 2.6% |
| Richmond | $759 | $931 |  | $557 | $713 | 2.8% |
| Rockland | $870 | $1,153 |  | $675 | $910 | 3.4% |
| Saratoga | $360 | $393 |  | $420 | $481 | 1.5% |
| Schenectady | $388 | $496 |  | $439 | $561 | 2.8% |
| Schoharie | $257 | $325 |  | $325 | $411 | 2.6% |
| Schuyler | $406 | $708 |  | $514 | $835 | 5.5% |
| Seneca | $438 | $512 |  | $529 | $618 | 1.7% |
| St. Lawrence | $398 | $501 |  | $486 | $592 | 2.2% |
| Steuben | $459 | $591 |  | $557 | $707 | 2.7% |
| Suffolk | $802 | $1,289 |  | $634 | $1,032 | 5.6% |
| Sullivan | $574 | $839 |  | $616 | $843 | 3.5% |
| Tioga | $373 | $557 |  | $478 | $690 | 4.2% |
| Tompkins | $318 | $361 |  | $374 | $444 | 1.9% |
| Ulster | $523 | $876 |  | $547 | $938 | 6.2% |
| Warren | $365 | $453 |  | $438 | $566 | 2.9% |
| Washington | $439 | $539 |  | $510 | $634 | 2.5% |
| Wayne | $544 | $615 |  | $637 | $654 | 0.3% |
| Westchester | $865 | $1,291 |  | $654 | $1,018 | 5.0% |
| Wyoming | $569 | $558 |  | $703 | $655 | -0.8% |
| Yates | $371 | $475 |  | $458 | $622 | 3.5% |

Notes: * Adjusted for geographic differences in prices (i.e., the cost of cost of providing services) and health status using the average CMS-HCC score for Medicare enrollees in each county.

**S1D Table. Per Capita HH Medicare Spending by County in New York, Unadjusted and Adjusted Spending levels in 2007 and 2016**

|  | **Unadjusted** | |  | **Adjusted Spending*** | | **Average Annual Percent Change in Adjusted Spending: 2007-2016** |
| --- | --- | --- | --- | --- | --- | --- |
| **County** | **2007** | **2016** |  | **2007** | **2016** |  |
| STATE TOTAL | $381 | $418 |  | $325 | $355 | 1.0% |
| Albany | $279 | $266 |  | $303 | $293 | -0.4% |
| Allegany | $288 | $368 |  | $349 | $436 | 2.5% |
| Bronx | $473 | $425 |  | $322 | $284 | -1.4% |
| Broome | $242 | $252 |  | $283 | $295 | 0.5% |
| Cattaraugus | $237 | $372 |  | $273 | $416 | 4.8% |
| Cayuga | $281 | $477 |  | $341 | $551 | 5.5% |
| Chautauqua | $280 | $412 |  | $318 | $469 | 4.4% |
| Chemung | $179 | $316 |  | $207 | $338 | 5.6% |
| Chenango | $55 | $150 |  | $68 | $180 | 11.3% |
| Clinton | $180 | $290 |  | $220 | $340 | 5.0% |
| Columbia | $210 | $198 |  | $242 | $225 | -0.8% |
| Cortland | $205 | $266 |  | $240 | $277 | 1.6% |
| Delaware | $179 | $185 |  | $225 | $225 | 0.0% |
| Dutchess | $244 | $285 |  | $236 | $256 | 0.9% |
| Erie | $353 | $491 |  | $375 | $467 | 2.5% |
| Essex | $124 | $261 |  | $154 | $334 | 9.0% |
| Franklin | $146 | $187 |  | $173 | $217 | 2.6% |
| Fulton | $269 | $280 |  | $307 | $305 | -0.1% |
| Genesee | $243 | $367 |  | $272 | $389 | 4.0% |
| Greene | $208 | $219 |  | $238 | $239 | 0.0% |
| Hamilton | $196 | $189 |  | $259 | $247 | -0.5% |
| Herkimer | $184 | $180 |  | $207 | $199 | -0.4% |
| Jefferson | $243 | $209 |  | $279 | $229 | -2.2% |
| Kings | $631 | $523 |  | $404 | $338 | -2.0% |
| Lewis | $98 | $144 |  | $123 | $174 | 3.9% |
| Livingston | $303 | $312 |  | $327 | $365 | 1.2% |
| Madison | $165 | $246 |  | $185 | $272 | 4.4% |
| Monroe | $473 | $479 |  | $491 | $488 | -0.1% |
| Montgomery | $335 | $365 |  | $372 | $398 | 0.7% |
| Nassau | $425 | $504 |  | $330 | $403 | 2.2% |
| New York | $529 | $495 |  | $405 | $400 | -0.1% |
| Niagara | $357 | $525 |  | $367 | $521 | 3.9% |
| Oneida | $194 | $186 |  | $221 | $197 | -1.2% |
| Onondaga | $369 | $469 |  | $388 | $484 | 2.5% |
| Ontario | $312 | $388 |  | $350 | $452 | 2.9% |
| Orange | $268 | $333 |  | $244 | $263 | 0.9% |
| Orleans | $189 | $383 |  | $221 | $413 | 7.2% |
| Oswego | $273 | $494 |  | $298 | $526 | 6.5% |
| Otsego | $181 | $245 |  | $235 | $312 | 3.2% |
| Putnam | $279 | $356 |  | $248 | $334 | 3.3% |
| Queens | $467 | $464 |  | $330 | $331 | 0.0% |
| Rensselaer | $270 | $267 |  | $281 | $292 | 0.4% |
| Richmond | $525 | $454 |  | $402 | $351 | -1.5% |
| Rockland | $283 | $361 |  | $229 | $287 | 2.6% |
| Saratoga | $258 | $326 |  | $298 | $395 | 3.2% |
| Schenectady | $223 | $269 |  | $246 | $300 | 2.2% |
| Schoharie | $131 | $285 |  | $167 | $365 | 9.0% |
| Schuyler | $148 | $233 |  | $187 | $274 | 4.3% |
| Seneca | $287 | $362 |  | $346 | $437 | 2.6% |
| St. Lawrence | $168 | $201 |  | $203 | $237 | 1.7% |
| Steuben | $157 | $288 |  | $188 | $346 | 7.0% |
| Suffolk | $338 | $470 |  | $279 | $378 | 3.4% |
| Sullivan | $178 | $177 |  | $197 | $187 | -0.6% |
| Tioga | $159 | $184 |  | $206 | $223 | 0.9% |
| Tompkins | $163 | $222 |  | $192 | $269 | 3.8% |
| Ulster | $257 | $288 |  | $272 | $319 | 1.8% |
| Warren | $233 | $285 |  | $276 | $350 | 2.7% |
| Washington | $260 | $324 |  | $305 | $376 | 2.4% |
| Wayne | $319 | $303 |  | $366 | $319 | -1.5% |
| Westchester | $421 | $450 |  | $335 | $359 | 0.8% |
| Wyoming | $248 | $274 |  | $310 | $324 | 0.5% |
| Yates | $172 | $267 |  | $221 | $346 | 5.1% |

Notes: * Adjusted for geographic differences in prices (i.e., the cost of cost of providing services) and health status using the average CMS-HCC score for Medicare enrollees in each county.

**S1E Table. Per Capita Outpatient Facility & ASC Medicare Spending by County in New York, Unadjusted and Adjusted Spending levels in 2007 and 2016**

|  | **Unadjusted** | |  | **Adjusted Spending*** | | **Average Annual Percent Change in Adjusted Spending: 2007-2016** |
| --- | --- | --- | --- | --- | --- | --- |
| **County** | **2007** | **2016** |  | **2007** | **2016** |  |
| STATE TOTAL | $743 | $1,464 |  | $677 | $1,256 | 7.1% |
| Albany | $617 | $1,156 |  | $661 | $1,186 | 6.7% |
| Allegany | $803 | $1,537 |  | $881 | $1,658 | 7.3% |
| Bronx | $734 | $1,356 |  | $551 | $942 | 6.1% |
| Broome | $897 | $1,926 |  | $1,018 | $2,037 | 8.0% |
| Cattaraugus | $710 | $1,276 |  | $752 | $1,314 | 6.4% |
| Cayuga | $611 | $1,152 |  | $688 | $1,187 | 6.3% |
| Chautauqua | $785 | $1,377 |  | $849 | $1,452 | 6.1% |
| Chemung | $837 | $1,724 |  | $912 | $1,719 | 7.3% |
| Chenango | $865 | $1,781 |  | $1,024 | $1,976 | 7.6% |
| Clinton | $1,165 | $2,185 |  | $1,226 | $2,129 | 6.3% |
| Columbia | $725 | $1,635 |  | $767 | $1,592 | 8.5% |
| Cortland | $729 | $1,487 |  | $791 | $1,449 | 7.0% |
| Delaware | $1,118 | $2,469 |  | $1,259 | $2,533 | 8.1% |
| Dutchess | $724 | $1,427 |  | $685 | $1,212 | 6.5% |
| Erie | $714 | $1,200 |  | $762 | $1,134 | 4.5% |
| Essex | $1,162 | $2,329 |  | $1,337 | $2,452 | 7.0% |
| Franklin | $1,055 | $2,090 |  | $1,131 | $2,133 | 7.3% |
| Fulton | $718 | $1,620 |  | $798 | $1,669 | 8.5% |
| Genesee | $670 | $1,856 |  | $698 | $1,810 | 11.2% |
| Greene | $588 | $1,428 |  | $635 | $1,361 | 8.8% |
| Hamilton | $928 | $1,385 |  | $1,131 | $1,689 | 4.6% |
| Herkimer | $875 | $1,578 |  | $937 | $1,640 | 6.4% |
| Jefferson | $804 | $1,581 |  | $846 | $1,572 | 7.1% |
| Kings | $662 | $1,326 |  | $461 | $892 | 7.6% |
| Lewis | $782 | $1,737 |  | $889 | $1,926 | 9.0% |
| Livingston | $785 | $1,902 |  | $832 | $2,035 | 10.5% |
| Madison | $738 | $1,286 |  | $835 | $1,409 | 6.0% |
| Monroe | $862 | $1,940 |  | $896 | $1,836 | 8.3% |
| Montgomery | $924 | $1,568 |  | $980 | $1,580 | 5.4% |
| Nassau | $591 | $1,416 |  | $489 | $1,148 | 10.0% |
| New York | $866 | $1,596 |  | $715 | $1,345 | 7.3% |
| Niagara | $913 | $1,463 |  | $943 | $1,438 | 4.8% |
| Oneida | $689 | $1,335 |  | $750 | $1,333 | 6.6% |
| Onondaga | $662 | $1,089 |  | $704 | $1,090 | 5.0% |
| Ontario | $784 | $1,780 |  | $875 | $1,937 | 9.2% |
| Orange | $868 | $1,351 |  | $756 | $1,089 | 4.1% |
| Orleans | $692 | $1,567 |  | $805 | $1,603 | 7.9% |
| Oswego | $742 | $1,268 |  | $829 | $1,311 | 5.2% |
| Otsego | $1,173 | $2,394 |  | $1,364 | $2,627 | 7.6% |
| Putnam | $907 | $1,510 |  | $861 | $1,355 | 5.2% |
| Queens | $622 | $1,253 |  | $477 | $928 | 7.7% |
| Rensselaer | $792 | $1,442 |  | $807 | $1,450 | 6.7% |
| Richmond | $882 | $1,476 |  | $779 | $1,197 | 4.9% |
| Rockland | $676 | $1,517 |  | $595 | $1,278 | 8.9% |
| Saratoga | $705 | $1,496 |  | $789 | $1,683 | 8.8% |
| Schenectady | $653 | $1,179 |  | $703 | $1,222 | 6.3% |
| Schoharie | $984 | $2,194 |  | $1,206 | $2,448 | 8.2% |
| Schuyler | $1,115 | $2,066 |  | $1,344 | $2,185 | 5.5% |
| Seneca | $781 | $1,835 |  | $904 | $2,034 | 9.4% |
| St. Lawrence | $1,021 | $2,213 |  | $1,148 | $2,315 | 8.1% |
| Steuben | $902 | $1,506 |  | $995 | $1,660 | 5.9% |
| Suffolk | $696 | $1,474 |  | $605 | $1,200 | 7.9% |
| Sullivan | $783 | $1,438 |  | $742 | $1,165 | 5.1% |
| Tioga | $759 | $1,601 |  | $950 | $1,819 | 7.5% |
| Tompkins | $708 | $1,388 |  | $828 | $1,597 | 7.6% |
| Ulster | $619 | $1,252 |  | $612 | $1,175 | 7.5% |
| Warren | $866 | $1,632 |  | $1,000 | $1,825 | 6.9% |
| Washington | $953 | $1,701 |  | $1,050 | $1,771 | 6.0% |
| Wayne | $709 | $1,715 |  | $805 | $1,681 | 8.5% |
| Westchester | $845 | $1,533 |  | $732 | $1,254 | 6.2% |
| Wyoming | $634 | $1,690 |  | $730 | $1,769 | 10.3% |
| Yates | $815 | $1,909 |  | $970 | $2,249 | 9.8% |

Notes: * Adjusted for geographic differences in prices (i.e., the cost of cost of providing services) and health status using the average CMS-HCC score for Medicare enrollees in each county.

**S1F Table. Per Capita E&M and Procedures Medicare Spending by County in New York, Unadjusted and Adjusted Spending levels in 2007 and 2016**

|  | **Unadjusted** | |  | **Adjusted Spending*** | | **Average Annual Percent Change in Adjusted Spending: 2007-2016** |
| --- | --- | --- | --- | --- | --- | --- |
| **County** | **2007** | **2016** |  | **2007** | **2016** |  |
| STATE TOTAL | $1,733 | $2,129 |  | $1,512 | $1,827 | 2.1% |
| Albany | $1,333 | $1,647 |  | $1,383 | $1,648 | 2.0% |
| Allegany | $896 | $1,105 |  | $986 | $1,204 | 2.2% |
| Bronx | $1,752 | $2,075 |  | $1,273 | $1,447 | 1.4% |
| Broome | $1,081 | $1,280 |  | $1,211 | $1,377 | 1.4% |
| Cattaraugus | $1,080 | $1,191 |  | $1,145 | $1,319 | 1.6% |
| Cayuga | $1,117 | $1,505 |  | $1,268 | $1,643 | 2.9% |
| Chautauqua | $1,038 | $1,400 |  | $1,095 | $1,487 | 3.5% |
| Chemung | $1,049 | $1,349 |  | $1,116 | $1,314 | 1.8% |
| Chenango | $871 | $1,152 |  | $1,014 | $1,306 | 2.9% |
| Clinton | $1,104 | $1,245 |  | $1,187 | $1,377 | 1.7% |
| Columbia | $1,291 | $1,618 |  | $1,324 | $1,667 | 2.6% |
| Cortland | $1,186 | $1,595 |  | $1,309 | $1,635 | 2.5% |
| Delaware | $920 | $1,155 |  | $1,014 | $1,305 | 2.8% |
| Dutchess | $1,579 | $1,959 |  | $1,575 | $1,852 | 1.8% |
| Erie | $1,045 | $1,362 |  | $1,127 | $1,428 | 2.7% |
| Essex | $864 | $985 |  | $990 | $1,127 | 1.4% |
| Franklin | $914 | $1,047 |  | $1,002 | $1,126 | 1.3% |
| Fulton | $1,107 | $1,488 |  | $1,186 | $1,477 | 2.5% |
| Genesee | $1,102 | $1,229 |  | $1,156 | $1,337 | 1.6% |
| Greene | $1,323 | $1,661 |  | $1,352 | $1,689 | 2.5% |
| Hamilton | $1,005 | $1,094 |  | $1,217 | $1,530 | 2.6% |
| Herkimer | $1,063 | $1,308 |  | $1,123 | $1,339 | 2.0% |
| Jefferson | $1,046 | $1,332 |  | $1,123 | $1,402 | 2.5% |
| Kings | $2,455 | $2,792 |  | $1,645 | $1,894 | 1.6% |
| Lewis | $885 | $1,260 |  | $1,011 | $1,343 | 3.2% |
| Livingston | $955 | $1,179 |  | $1,015 | $1,295 | 2.7% |
| Madison | $1,131 | $1,391 |  | $1,328 | $1,591 | 2.0% |
| Monroe | $1,005 | $1,348 |  | $1,029 | $1,273 | 2.4% |
| Montgomery | $1,136 | $1,403 |  | $1,167 | $1,408 | 2.1% |
| Nassau | $2,450 | $2,776 |  | $1,961 | $2,295 | 1.8% |
| New York | $1,921 | $2,290 |  | $1,576 | $1,941 | 2.3% |
| Niagara | $1,120 | $1,362 |  | $1,182 | $1,430 | 2.1% |
| Oneida | $1,165 | $1,473 |  | $1,236 | $1,534 | 2.4% |
| Onondaga | $1,199 | $1,563 |  | $1,318 | $1,674 | 2.7% |
| Ontario | $961 | $1,265 |  | $1,057 | $1,374 | 3.0% |
| Orange | $1,674 | $2,092 |  | $1,514 | $1,886 | 2.5% |
| Orleans | $907 | $1,218 |  | $1,045 | $1,274 | 2.2% |
| Oswego | $1,175 | $1,481 |  | $1,329 | $1,624 | 2.3% |
| Otsego | $787 | $1,082 |  | $944 | $1,262 | 3.3% |
| Putnam | $1,829 | $2,056 |  | $1,814 | $1,990 | 1.0% |
| Queens | $2,123 | $2,559 |  | $1,618 | $1,935 | 2.0% |
| Rensselaer | $1,330 | $1,610 |  | $1,332 | $1,621 | 2.2% |
| Richmond | $1,956 | $2,424 |  | $1,591 | $2,003 | 2.6% |
| Rockland | $2,172 | $2,608 |  | $1,877 | $2,215 | 1.9% |
| Saratoga | $1,207 | $1,482 |  | $1,329 | $1,646 | 2.4% |
| Schenectady | $1,218 | $1,493 |  | $1,296 | $1,524 | 1.8% |
| Schoharie | $908 | $1,137 |  | $1,077 | $1,325 | 2.3% |
| Schuyler | $842 | $1,098 |  | $995 | $1,153 | 1.7% |
| Seneca | $992 | $1,312 |  | $1,139 | $1,463 | 2.8% |
| St. Lawrence | $952 | $1,125 |  | $1,067 | $1,216 | 1.5% |
| Steuben | $923 | $1,154 |  | $1,035 | $1,285 | 2.4% |
| Suffolk | $2,169 | $2,677 |  | $1,843 | $2,243 | 2.2% |
| Sullivan | $1,421 | $1,774 |  | $1,367 | $1,736 | 2.7% |
| Tioga | $977 | $1,232 |  | $1,197 | $1,437 | 2.1% |
| Tompkins | $1,026 | $1,258 |  | $1,244 | $1,579 | 2.7% |
| Ulster | $1,445 | $1,818 |  | $1,443 | $1,825 | 2.6% |
| Warren | $1,045 | $1,236 |  | $1,159 | $1,303 | 1.3% |
| Washington | $1,056 | $1,189 |  | $1,138 | $1,273 | 1.2% |
| Wayne | $874 | $1,303 |  | $990 | $1,290 | 3.0% |
| Westchester | $2,030 | $2,378 |  | $1,710 | $1,994 | 1.7% |
| Wyoming | $942 | $1,211 |  | $1,081 | $1,401 | 2.9% |
| Yates | $833 | $1,016 |  | $964 | $1,093 | 1.4% |

Notes: * Adjusted for geographic differences in prices (i.e., the cost of cost of providing services) and health status using the average CMS-HCC score for Medicare enrollees in each county.

**S1G Table. Per Capita Imaging and Tests Medicare Spending by County in New York, Unadjusted and Adjusted Spending levels in 2007 and 2016**

|  | **Unadjusted** | |  | **Adjusted Spending*** | | **Average Annual Percent Change in Adjusted Spending: 2007-2016** |
| --- | --- | --- | --- | --- | --- | --- |
| **County** | **2007** | **2016** |  | **2007** | **2016** |  |
| STATE TOTAL | $726 | $738 |  | $659 | $647 | -0.2% |
| Albany | $503 | $422 |  | $549 | $419 | -3.0% |
| Allegany | $285 | $279 |  | $327 | $301 | -0.9% |
| Bronx | $675 | $664 |  | $514 | $485 | -0.6% |
| Broome | $297 | $287 |  | $350 | $295 | -1.9% |
| Cattaraugus | $391 | $356 |  | $434 | $363 | -2.0% |
| Cayuga | $429 | $480 |  | $510 | $503 | -0.1% |
| Chautauqua | $330 | $344 |  | $354 | $360 | 0.2% |
| Chemung | $332 | $303 |  | $360 | $293 | -2.3% |
| Chenango | $249 | $236 |  | $305 | $257 | -1.9% |
| Clinton | $339 | $286 |  | $377 | $302 | -2.4% |
| Columbia | $479 | $395 |  | $509 | $398 | -2.7% |
| Cortland | $352 | $401 |  | $400 | $387 | -0.4% |
| Delaware | $248 | $265 |  | $279 | $285 | 0.3% |
| Dutchess | $696 | $607 |  | $722 | $576 | -2.5% |
| Erie | $416 | $457 |  | $468 | $459 | -0.2% |
| Essex | $270 | $235 |  | $326 | $271 | -2.1% |
| Franklin | $238 | $221 |  | $271 | $234 | -1.6% |
| Fulton | $285 | $315 |  | $313 | $314 | 0.0% |
| Genesee | $340 | $340 |  | $383 | $333 | -1.6% |
| Greene | $583 | $425 |  | $627 | $410 | -4.6% |
| Hamilton | $430 | $355 |  | $535 | $426 | -2.5% |
| Herkimer | $422 | $436 |  | $470 | $455 | -0.3% |
| Jefferson | $397 | $392 |  | $443 | $404 | -1.0% |
| Kings | $1,092 | $1,125 |  | $769 | $795 | 0.4% |
| Lewis | $261 | $318 |  | $308 | $353 | 1.5% |
| Livingston | $335 | $293 |  | $385 | $315 | -2.2% |
| Madison | $450 | $421 |  | $552 | $466 | -1.9% |
| Monroe | $373 | $314 |  | $408 | $294 | -3.6% |
| Montgomery | $342 | $307 |  | $364 | $304 | -2.0% |
| Nassau | $1,124 | $1,067 |  | $945 | $929 | -0.2% |
| New York | $828 | $800 |  | $705 | $710 | 0.1% |
| Niagara | $341 | $405 |  | $369 | $422 | 1.5% |
| Oneida | $494 | $525 |  | $556 | $528 | -0.6% |
| Onondaga | $597 | $583 |  | $687 | $598 | -1.5% |
| Ontario | $249 | $261 |  | $291 | $280 | -0.4% |
| Orange | $734 | $680 |  | $694 | $618 | -1.3% |
| Orleans | $264 | $325 |  | $321 | $322 | 0.0% |
| Oswego | $416 | $465 |  | $492 | $491 | 0.0% |
| Otsego | $191 | $207 |  | $231 | $238 | 0.3% |
| Putnam | $718 | $701 |  | $735 | $684 | -0.8% |
| Queens | $963 | $989 |  | $767 | $773 | 0.1% |
| Rensselaer | $432 | $407 |  | $450 | $400 | -1.3% |
| Richmond | $880 | $929 |  | $750 | $780 | 0.4% |
| Rockland | $894 | $917 |  | $817 | $807 | -0.1% |
| Saratoga | $445 | $413 |  | $515 | $448 | -1.6% |
| Schenectady | $388 | $397 |  | $423 | $398 | -0.7% |
| Schoharie | $241 | $247 |  | $292 | $282 | -0.4% |
| Schuyler | $234 | $256 |  | $276 | $274 | -0.1% |
| Seneca | $284 | $295 |  | $335 | $326 | -0.3% |
| St. Lawrence | $224 | $231 |  | $257 | $248 | -0.4% |
| Steuben | $266 | $263 |  | $305 | $284 | -0.8% |
| Suffolk | $961 | $1,003 |  | $855 | $887 | 0.4% |
| Sullivan | $582 | $547 |  | $578 | $509 | -1.4% |
| Tioga | $290 | $304 |  | $365 | $329 | -1.2% |
| Tompkins | $314 | $317 |  | $377 | $366 | -0.3% |
| Ulster | $586 | $571 |  | $611 | $565 | -0.9% |
| Warren | $442 | $336 |  | $520 | $365 | -3.9% |
| Washington | $363 | $301 |  | $416 | $306 | -3.3% |
| Wayne | $249 | $287 |  | $297 | $279 | -0.7% |
| Westchester | $809 | $710 |  | $715 | $616 | -1.6% |
| Wyoming | $293 | $317 |  | $356 | $348 | -0.3% |
| Yates | $223 | $226 |  | $275 | $268 | -0.3% |

Notes: * Adjusted for geographic differences in prices (i.e., the cost of cost of providing services) and health status using the average CMS-HCC score for Medicare enrollees in each county.

**S1H Table. Per Capita DME Medicare Spending by County in New York, Unadjusted and Adjusted Spending levels in 2007 and 2016**

|  | **Unadjusted** | |  | **Adjusted Spending*** | | **Average Annual Percent Change in Adjusted Spending: 2007-2016** |
| --- | --- | --- | --- | --- | --- | --- |
| **County** | **2007** | **2016** |  | **2007** | **2016** |  |
| STATE TOTAL | $191 | $147 |  | $180 | $144 | -2.4% |
| Albany | $186 | $146 |  | $181 | $152 | -1.9% |
| Allegany | $234 | $179 |  | $242 | $183 | -3.0% |
| Bronx | $177 | $141 |  | $146 | $119 | -2.2% |
| Broome | $219 | $154 |  | $228 | $156 | -4.1% |
| Cattaraugus | $250 | $195 |  | $249 | $189 | -3.0% |
| Cayuga | $221 | $192 |  | $234 | $193 | -2.1% |
| Chautauqua | $229 | $196 |  | $225 | $194 | -1.7% |
| Chemung | $239 | $202 |  | $237 | $193 | -2.3% |
| Chenango | $249 | $180 |  | $271 | $188 | -3.9% |
| Clinton | $228 | $211 |  | $230 | $215 | -0.8% |
| Columbia | $179 | $139 |  | $183 | $141 | -2.9% |
| Cortland | $243 | $198 |  | $249 | $187 | -3.1% |
| Delaware | $230 | $173 |  | $245 | $186 | -3.0% |
| Dutchess | $165 | $140 |  | $169 | $147 | -1.6% |
| Erie | $203 | $150 |  | $205 | $158 | -2.8% |
| Essex | $206 | $171 |  | $224 | $189 | -1.8% |
| Franklin | $248 | $219 |  | $256 | $223 | -1.5% |
| Fulton | $259 | $229 |  | $259 | $217 | -1.9% |
| Genesee | $232 | $175 |  | $227 | $163 | -3.6% |
| Greene | $172 | $156 |  | $174 | $150 | -1.6% |
| Hamilton | $159 | $133 |  | $182 | $155 | -1.8% |
| Herkimer | $219 | $157 |  | $217 | $162 | -3.2% |
| Jefferson | $248 | $213 |  | $249 | $216 | -1.6% |
| Kings | $238 | $161 |  | $183 | $131 | -3.6% |
| Lewis | $218 | $157 |  | $233 | $165 | -3.8% |
| Livingston | $218 | $161 |  | $217 | $181 | -2.0% |
| Madison | $214 | $156 |  | $233 | $185 | -2.6% |
| Monroe | $219 | $167 |  | $209 | $161 | -2.9% |
| Montgomery | $213 | $188 |  | $205 | $178 | -1.6% |
| Nassau | $175 | $139 |  | $160 | $140 | -1.5% |
| New York | $153 | $108 |  | $142 | $111 | -2.7% |
| Niagara | $219 | $156 |  | $216 | $170 | -2.6% |
| Oneida | $202 | $187 |  | $201 | $184 | -0.9% |
| Onondaga | $203 | $163 |  | $208 | $178 | -1.7% |
| Ontario | $172 | $153 |  | $176 | $170 | -0.4% |
| Orange | $197 | $154 |  | $185 | $156 | -1.9% |
| Orleans | $217 | $150 |  | $234 | $156 | -4.4% |
| Oswego | $261 | $189 |  | $276 | $215 | -2.7% |
| Otsego | $201 | $154 |  | $226 | $171 | -3.1% |
| Putnam | $168 | $102 |  | $178 | $115 | -4.8% |
| Queens | $172 | $134 |  | $147 | $121 | -2.1% |
| Rensselaer | $195 | $137 |  | $183 | $143 | -2.7% |
| Richmond | $170 | $129 |  | $159 | $128 | -2.3% |
| Rockland | $135 | $126 |  | $132 | $129 | -0.2% |
| Saratoga | $203 | $145 |  | $210 | $165 | -2.7% |
| Schenectady | $194 | $170 |  | $192 | $179 | -0.8% |
| Schoharie | $237 | $142 |  | $267 | $176 | -4.6% |
| Schuyler | $235 | $187 |  | $255 | $192 | -3.1% |
| Seneca | $207 | $218 |  | $220 | $230 | 0.5% |
| St. Lawrence | $248 | $230 |  | $261 | $236 | -1.1% |
| Steuben | $224 | $176 |  | $233 | $183 | -2.7% |
| Suffolk | $181 | $139 |  | $175 | $143 | -2.2% |
| Sullivan | $219 | $197 |  | $217 | $187 | -1.6% |
| Tioga | $212 | $170 |  | $242 | $182 | -3.1% |
| Tompkins | $233 | $145 |  | $263 | $167 | -4.9% |
| Ulster | $186 | $156 |  | $188 | $161 | -1.7% |
| Warren | $197 | $149 |  | $205 | $158 | -2.8% |
| Washington | $220 | $162 |  | $222 | $162 | -3.4% |
| Wayne | $227 | $171 |  | $240 | $171 | -3.7% |
| Westchester | $133 | $111 |  | $127 | $113 | -1.3% |
| Wyoming | $196 | $175 |  | $211 | $183 | -1.6% |
| Yates | $198 | $134 |  | $215 | $155 | -3.6% |

Notes: * Adjusted for geographic differences in prices (i.e., the cost of cost of providing services) and health status using the average CMS-HCC score for Medicare enrollees in each county.

**S1I Table. Per Capita Ambulance Medicare Spending by County in New York, Unadjusted and Adjusted Spending levels in 2007 and 2016**

|  | **Unadjusted** | |  | **Adjusted Spending*** | | **Average Annual Percent Change in Adjusted Spending: 2007-2016** |
| --- | --- | --- | --- | --- | --- | --- |
| **County** | **2007** | **2016** |  | **2007** | **2016** |  |
| STATE TOTAL | $111 | $127 |  | $97 | $119 | 2.4% |
| Albany | $110 | $129 |  | $113 | $137 | 2.2% |
| Allegany | $127 | $158 |  | $134 | $133 | -0.1% |
| Bronx | $184 | $163 |  | $129 | $133 | 0.3% |
| Broome | $87 | $112 |  | $96 | $121 | 2.6% |
| Cattaraugus | $150 | $153 |  | $155 | $131 | -1.9% |
| Cayuga | $82 | $139 |  | $91 | $142 | 5.0% |
| Chautauqua | $86 | $145 |  | $88 | $136 | 4.9% |
| Chemung | $91 | $137 |  | $95 | $138 | 4.2% |
| Chenango | $90 | $134 |  | $103 | $120 | 1.7% |
| Clinton | $102 | $120 |  | $108 | $120 | 1.1% |
| Columbia | $153 | $155 |  | $151 | $131 | -1.6% |
| Cortland | $94 | $133 |  | $101 | $128 | 2.7% |
| Delaware | $104 | $148 |  | $110 | $115 | 0.5% |
| Dutchess | $109 | $139 |  | $107 | $131 | 2.4% |
| Erie | $86 | $104 |  | $92 | $110 | 2.0% |
| Essex | $92 | $107 |  | $97 | $92 | -0.5% |
| Franklin | $81 | $133 |  | $86 | $118 | 3.5% |
| Fulton | $125 | $188 |  | $136 | $168 | 2.3% |
| Genesee | $131 | $159 |  | $133 | $136 | 0.2% |
| Greene | $132 | $170 |  | $131 | $130 | -0.1% |
| Hamilton | $69 | $118 |  | $80 | $93 | 1.8% |
| Herkimer | $153 | $163 |  | $159 | $147 | -0.9% |
| Jefferson | $88 | $122 |  | $92 | $117 | 2.7% |
| Kings | $186 | $227 |  | $122 | $187 | 4.9% |
| Lewis | $62 | $132 |  | $66 | $109 | 5.7% |
| Livingston | $98 | $130 |  | $103 | $115 | 1.2% |
| Madison | $91 | $112 |  | $104 | $113 | 0.9% |
| Monroe | $118 | $150 |  | $119 | $152 | 2.7% |
| Montgomery | $147 | $167 |  | $148 | $149 | 0.1% |
| Nassau | $85 | $84 |  | $67 | $80 | 2.0% |
| New York | $94 | $89 |  | $74 | $91 | 2.3% |
| Niagara | $83 | $82 |  | $87 | $88 | 0.0% |
| Oneida | $113 | $145 |  | $119 | $147 | 2.4% |
| Onondaga | $87 | $123 |  | $94 | $135 | 4.1% |
| Ontario | $101 | $130 |  | $108 | $141 | 3.0% |
| Orange | $133 | $129 |  | $120 | $119 | 0.0% |
| Orleans | $99 | $190 |  | $111 | $158 | 4.0% |
| Oswego | $105 | $145 |  | $116 | $132 | 1.5% |
| Otsego | $45 | $97 |  | $55 | $97 | 6.6% |
| Putnam | $85 | $103 |  | $85 | $101 | 2.0% |
| Queens | $106 | $128 |  | $80 | $112 | 3.9% |
| Rensselaer | $130 | $149 |  | $127 | $152 | 2.0% |
| Richmond | $109 | $132 |  | $87 | $126 | 4.2% |
| Rockland | $144 | $148 |  | $123 | $132 | 0.9% |
| Saratoga | $93 | $125 |  | $102 | $135 | 3.2% |
| Schenectady | $105 | $132 |  | $111 | $141 | 2.7% |
| Schoharie | $66 | $106 |  | $76 | $103 | 3.5% |
| Schuyler | $83 | $119 |  | $94 | $108 | 1.6% |
| Seneca | $101 | $145 |  | $115 | $141 | 2.3% |
| St. Lawrence | $112 | $153 |  | $123 | $136 | 1.2% |
| Steuben | $91 | $145 |  | $99 | $133 | 3.3% |
| Suffolk | $74 | $71 |  | $62 | $66 | 0.7% |
| Sullivan | $158 | $166 |  | $151 | $132 | -1.4% |
| Tioga | $60 | $61 |  | $72 | $64 | -1.4% |
| Tompkins | $84 | $96 |  | $99 | $118 | 2.0% |
| Ulster | $120 | $141 |  | $117 | $137 | 1.7% |
| Warren | $99 | $127 |  | $109 | $135 | 2.4% |
| Washington | $128 | $159 |  | $135 | $141 | 0.5% |
| Wayne | $102 | $180 |  | $114 | $157 | 3.6% |
| Westchester | $110 | $129 |  | $91 | $120 | 3.1% |
| Wyoming | $71 | $119 |  | $78 | $115 | 4.4% |
| Yates | $80 | $123 |  | $90 | $130 | 4.2% |

Notes: * Adjusted for geographic differences in prices (i.e., the cost of cost of providing services) and health status using the average CMS-HCC score for Medicare enrollees in each county.

**S1J Table. Per Capita Part B Drugs Medicare Spending by County in New York, Unadjusted and Adjusted Spending levels in 2007 and 2016**

|  | **Unadjusted** | |  | **Adjusted Spending*** | | **Average Annual Percent Change in Adjusted Spending: 2007-2016** |
| --- | --- | --- | --- | --- | --- | --- |
| **County** | **2007** | **2016** |  | **2007** | **2016** |  |
| STATE TOTAL | $256 | $437 |  | $239 | $412 | 6.2% |
| Albany | $294 | $671 |  | $285 | $660 | 9.8% |
| Allegany | $260 | $341 |  | $268 | $357 | 3.2% |
| Bronx | $210 | $256 |  | $172 | $207 | 2.1% |
| Broome | $399 | $376 |  | $416 | $381 | -1.0% |
| Cattaraugus | $277 | $502 |  | $275 | $498 | 6.8% |
| Cayuga | $355 | $547 |  | $373 | $562 | 4.6% |
| Chautauqua | $231 | $483 |  | $226 | $489 | 8.9% |
| Chemung | $231 | $353 |  | $229 | $336 | 4.4% |
| Chenango | $280 | $305 |  | $304 | $330 | 0.9% |
| Clinton | $103 | $277 |  | $104 | $290 | 12.1% |
| Columbia | $260 | $492 |  | $265 | $505 | 7.4% |
| Cortland | $327 | $408 |  | $334 | $414 | 2.4% |
| Delaware | $212 | $258 |  | $225 | $282 | 2.5% |
| Dutchess | $273 | $509 |  | $278 | $501 | 6.8% |
| Erie | $223 | $499 |  | $223 | $491 | 9.2% |
| Essex | $100 | $359 |  | $108 | $405 | 15.8% |
| Franklin | $162 | $240 |  | $167 | $250 | 4.6% |
| Fulton | $234 | $401 |  | $234 | $396 | 6.0% |
| Genesee | $184 | $309 |  | $178 | $295 | 5.8% |
| Greene | $276 | $565 |  | $279 | $550 | 7.8% |
| Hamilton | $116 | $394 |  | $133 | $464 | 14.9% |
| Herkimer | $193 | $516 |  | $191 | $539 | 12.2% |
| Jefferson | $274 | $507 |  | $274 | $513 | 7.2% |
| Kings | $252 | $265 |  | $192 | $208 | 0.9% |
| Lewis | $266 | $509 |  | $283 | $561 | 7.9% |
| Livingston | $111 | $241 |  | $110 | $260 | 10.0% |
| Madison | $217 | $503 |  | $236 | $553 | 9.9% |
| Monroe | $129 | $154 |  | $122 | $146 | 2.0% |
| Montgomery | $258 | $376 |  | $248 | $378 | 4.8% |
| Nassau | $339 | $512 |  | $309 | $490 | 5.3% |
| New York | $208 | $322 |  | $190 | $311 | 5.6% |
| Niagara | $217 | $470 |  | $213 | $477 | 9.4% |
| Oneida | $266 | $716 |  | $263 | $701 | 11.5% |
| Onondaga | $327 | $686 |  | $334 | $695 | 8.5% |
| Ontario | $161 | $208 |  | $165 | $220 | 3.3% |
| Orange | $226 | $483 |  | $211 | $458 | 9.0% |
| Orleans | $204 | $255 |  | $219 | $245 | 1.2% |
| Oswego | $361 | $710 |  | $380 | $739 | 7.7% |
| Otsego | $96 | $116 |  | $108 | $131 | 2.2% |
| Putnam | $293 | $552 |  | $308 | $570 | 7.1% |
| Queens | $268 | $383 |  | $227 | $330 | 4.2% |
| Rensselaer | $221 | $591 |  | $206 | $578 | 12.1% |
| Richmond | $216 | $316 |  | $200 | $295 | 4.4% |
| Rockland | $252 | $438 |  | $245 | $421 | 6.2% |
| Saratoga | $222 | $490 |  | $229 | $523 | 9.6% |
| Schenectady | $286 | $601 |  | $283 | $596 | 8.6% |
| Schoharie | $123 | $368 |  | $138 | $430 | 13.5% |
| Schuyler | $178 | $217 |  | $193 | $228 | 1.9% |
| Seneca | $214 | $311 |  | $227 | $332 | 4.3% |
| St. Lawrence | $117 | $180 |  | $123 | $190 | 4.9% |
| Steuben | $196 | $281 |  | $204 | $306 | 4.6% |
| Suffolk | $313 | $660 |  | $301 | $639 | 8.7% |
| Sullivan | $175 | $365 |  | $173 | $350 | 8.1% |
| Tioga | $544 | $323 |  | $618 | $342 | -6.4% |
| Tompkins | $235 | $237 |  | $264 | $273 | 0.4% |
| Ulster | $226 | $504 |  | $229 | $520 | 9.6% |
| Warren | $135 | $227 |  | $140 | $241 | 6.2% |
| Washington | $158 | $304 |  | $159 | $302 | 7.4% |
| Wayne | $177 | $165 |  | $186 | $165 | -1.3% |
| Westchester | $251 | $446 |  | $239 | $426 | 6.6% |
| Wyoming | $125 | $362 |  | $134 | $385 | 12.5% |
| Yates | $169 | $417 |  | $184 | $480 | 11.3% |

Notes: * Adjusted for geographic differences in prices (i.e., the cost of cost of providing services) and health status using the average CMS-HCC score for Medicare enrollees in each county.
